# Representation and reporting of patients with cancer in mortality-focused adult ICU randomized trials: a systematic review

**DOI:** 10.1186/s12871-026-03840-w

**Published:** 2026-04-20

**Authors:** Rafael Hortêncio Melo, Luciana Gioli-Pereira, Mariana Resende Bustamante, Gustavo Potratz Gonçalves, Valéria Urresti Orias, Amanda Pascoal Valle Felício, Thiago Domingos Corrêa, Rogério Da Hora Passos

**Affiliations:** 1 Hospital Municipal Gilson de Cássia Marques de Carvalho, Hospital Israelita Albert Einstein, São Paulo, SP Brazil; 2 Faculdade Israelita de Ciências da Saúde Albert Einstein, Hospital Israelita Albert Einstein, São Paulo, SP Brazil; 3 Hospital Israelita Albert Einstein, São Paulo, SP Brazil

**Keywords:** Critical care, Oncology, Randomized controlled trials, Mortality

## Abstract

**Background:**

Patients with cancer represent a substantial and growing proportion of intensive care unit (ICU) admissions. However, how consistently they are explicitly included, reported, and analytically represented in randomized controlled trials (RCTs) of ICU interventions remains unclear.

**Methods:**

We conducted a systematic review of adult ICU RCTs published from January 1, 2000 through the date of the final database search (January 19, 2026). Eligible studies enrolled adult patients (≥ 18 years) managed as critically ill, prespecified all-cause mortality as a primary or co-primary outcome, included at least 100 participants, and were published in English-language journals indexed in the National Library of Medicine Core Clinical Journals subset. Two independent reviewers screened records and extracted trial characteristics, intervention domains, and reporting of cancer status. Trials were classified according to whether patients with cancer were explicitly included, explicitly excluded, or not reported. Interventions were grouped into five domains: pharmacologic/immunomodulatory therapies, hemodynamic/resuscitation strategies, mechanical ventilation/oxygenation, renal/metabolic support, and general ICU management.

**Results:**

A total of 77 RCTs comprising 144,548 critically ill adults met the inclusion criteria. Twenty-four trials (31.1%) explicitly reported inclusion of patients with cancer, nine (11.6%) explicitly excluded them, and 44 (57.1%) did not report cancer status. Among studies reporting prevalence, the proportion of enrolled patients with cancer ranged from 3% to 33%, with only four trials including ≥ 20% oncology patients. Inclusion varied across intervention domains, ranging from 18.2% in general ICU management trials to 40% in pharmacologic or immunomodulatory intervention trials. None of the included studies reported mortality outcomes stratified according to cancer status.

**Conclusions:**

In adult ICU randomized trials that prespecified mortality as a primary or co-primary outcome, cancer status was frequently not reported and explicit inclusion of patients with cancer was limited. The absence of oncology-specific outcome reporting restricts the ability to assess the applicability of trial findings to this growing ICU population. Future ICU trials should report cancer status more consistently and consider prespecified oncology-focused analyses when clinically justified.

**Supplementary Information:**

The online version contains supplementary material available at 10.1186/s12871-026-03840-w.

## Introduction

Advances in cancer diagnosis and treatment over recent decades have markedly improved survival among patients with both solid and hematologic malignancies [1–3]. As a consequence, an increasing number of patients with cancer are living longer and presenting to intensive care units (ICUs) with acute complications related either to the malignancy itself or to cancer-directed therapies [4–6]. These complications commonly include sepsis, respiratory failure, organ dysfunction, and treatment-related toxicities that frequently require advanced organ support.

In several contemporary cohorts, patients with cancer represent a substantial proportion of ICU admissions. Some multicenter studies have reported that individuals with malignancy account for approximately 17–21% of ICU admissions in selected settings, particularly in populations admitted for septic shock or acute respiratory distress syndrome (ARDS) [4, 7]. However, these estimates vary widely depending on geographic region, cancer subtype distribution, ICU case mix, and admission policies. Despite this variability, the growing presence of patients with cancer in critical care environments has been consistently documented.

At the same time, randomized controlled trials (RCTs) remain the cornerstone of evidence guiding ICU practice. Many contemporary guidelines in critical care rely heavily on results from large RCTs evaluating interventions such as ventilation strategies, hemodynamic management, pharmacologic therapies, and organ support techniques. The extent to which these trials represent real-world ICU populations is therefore central to their external validity and clinical applicability.

Prior evaluations in selected clinical domains have raised concerns that patients with cancer may be underrepresented in clinical trials conducted in critically ill populations. For example, analyses of sepsis trials have suggested that malignancy is frequently listed among exclusion criteria or indirectly excluded through terms such as “terminal illness” or “severe immunosuppression” [8–10]. However, it remains unclear whether similar patterns occur across the broader spectrum of ICU intervention trials. Moreover, little attention has been paid to how often cancer status is explicitly reported in trial populations or whether outcomes are analyzed according to oncologic status.

This question is also relevant to anesthesiology and perioperative critical care. In many health systems, anesthesiologists play a central role in ICU management, perioperative critical care, airway and ventilatory support, hemodynamic stabilization, and postoperative rescue pathways. Patients with malignancy frequently undergo major surgical procedures, receive complex oncologic therapies, and experience treatment-related complications that require critical care support. Understanding whether these patients are represented and transparently reported in ICU randomized trials therefore has direct implications for anesthesiology-led critical care and perioperative practice [11, 12].

The objective of this systematic review was to characterize how patients with cancer are reported, explicitly included or excluded, and analytically represented in adult ICU randomized controlled trials that prespecify all-cause mortality as a primary or co-primary outcome.

## Methods

### Study design and reporting

This systematic review was conducted according to the methodological guidance of the Cochrane Handbook for Systematic Reviews of Interventions and is reported following the Preferred Reporting Items for Systematic Reviews and Meta-Analyses (PRISMA 2020) statement [13, 14]. The study protocol was prospectively registered in the International Prospective Register of Systematic Reviews (PROSPERO) (registration number CRD420251129171). Because the study involved analysis of previously published data, institutional review board approval and informed consent were not required. 

### Eligibility criteria

Randomized controlled trials were eligible if they met al.l of the following criteria: publication between January 1, 2000 and the date of the final database search (January 19, 2026); enrollment of adult patients aged 18 years or older; study populations managed in intensive care units or otherwise defined as critically ill; prespecification of all-cause mortality as a primary or co-primary outcome; and a total sample size of at least 100 participants. Eligible studies were required to be published in English-language journals indexed in the National Library of Medicine Core Clinical Journals subset [15].

Restriction to Core Clinical Journals was prespecified to focus the review on large and clinically influential ICU randomized trials that are most likely to inform international guidelines and broadly influence critical care practice. This approach prioritizes trials with high clinical visibility but may exclude relevant studies published in subspecialty journals.

Studies were excluded if they enrolled pediatric populations, were conference abstracts, editorials, narrative reviews, or case reports, involved preclinical or animal models, or reported mortality only as part of a composite endpoint without separate analysis. 

### Search strategy

A systematic search of PubMed, Embase, and the Cochrane Central Register of Controlled Trials (CENTRAL) was performed from database inception through January 2026. The search strategy combined controlled vocabulary terms (Medical Subject Headings and Emtree) with free-text keywords related to intensive care, critical illness, randomized controlled trials, and mortality outcomes.The complete reproducible search strategies for each database, including Boolean operators, filters, and limits applied, are provided in the Supplementary Appendix. 

### Study selection

Two investigators (R.H.M. and L.G.P.) independently screened titles and abstracts to identify potentially eligible studies. Full-text articles were then independently assessed for inclusion according to the predefined eligibility criteria. Disagreements were resolved through discussion, and when consensus could not be reached, a third investigator (R.D.H.P.) adjudicated the decision. 

### Data extraction

Two reviewers independently extracted data from each included trial using a standardized data collection form. Extracted variables included publication year, sample size, mean or median patient age, sex distribution, ICU population or primary clinical condition, type of intervention, intervention domain, and reporting of cancer status. When available, the proportion of enrolled patients with cancer was also recorded. 

### Definition of cancer status

For the purposes of this review, patients with cancer were defined as participants described in the trial report as having active solid malignancy, hematologic malignancy, metastatic disease, or cancer-related immunosuppression. A remote history of cancer in remission was not classified as active malignancy unless the trial explicitly categorized such patients as having malignancy within eligibility criteria or baseline characteristics. Because definitions of malignancy varied across individual trials, harmonization according to cancer subtype, stage, or treatment status was not consistently feasible. 

### Classification of cancer status in trials

Studies were categorized according to whether patients with cancer were explicitly included, explicitly excluded, or not reported, using prespecified decision rules to ensure consistent classification. A study was considered included when eligibility criteria explicitly allowed enrollment of patients with malignancy or when baseline characteristics tables documented participants with cancer. Exclusion was assigned when malignancy appeared among the exclusion criteria, including specifications such as active cancer, metastatic disease, or hematologic malignancy. Studies excluding only specific cancer subgroups (e.g., metastatic disease while allowing localized cancer) were also categorized as excluded in the primary analysis, as cancer-related eligibility restrictions were present. The not reported category was used when cancer status was not mentioned in eligibility criteria, baseline characteristics tables, flow diagrams, or supplementary materials. Lack of reporting was not interpreted as absence of cancer among enrolled participants. Ambiguous terms such as terminal illness, poor prognosis, immunosuppression, or limited life expectancy were not considered evidence of malignancy exclusion unless cancer was explicitly specified. 

### Intervention domains

To facilitate interpretation, interventions were grouped into five predefined domains according to the primary target of the intervention. These domains included pharmacologic or immunomodulatory therapies, hemodynamic or resuscitation strategies, mechanical ventilation or oxygenation strategies, renal or metabolic support interventions, and general ICU management or miscellaneous interventions. Domain classification was performed independently by two reviewers, and inter-reviewer agreement was assessed using Cohen’s kappa coefficient. 

### Risk of bias assessment

Risk of bias for each included randomized controlled trial was evaluated using the Cochrane Risk of Bias 2 (RoB-2) tool. Two reviewers independently assessed the following domains: randomization process, deviations from intended interventions, missing outcome data, measurement of the outcome, and selection of the reported result. Disagreements were resolved by consensus. 

### Data synthesis

Because the objective of this study was descriptive, results were summarized using descriptive statistics. We reported the frequency and proportion of trials classified as including, excluding, or not reporting cancer status. When available, the proportion of enrolled patients with cancer was summarized descriptively. Temporal trends in reporting of cancer status were explored descriptively across the study period. To facilitate a clear comparison between earlier and more contemporary trials, analyses were performed using two broader time periods (2000–2019 and 2020–2025), rather than finer temporal stratification.

## Results

The literature search identified 6,810 records. After removal of duplicates and screening of titles and abstracts, full-text assessment was performed for potentially eligible studies. A total of 77 randomized controlled trials met the inclusion criteria and were included in the final analysis.

**Fig. 1:**
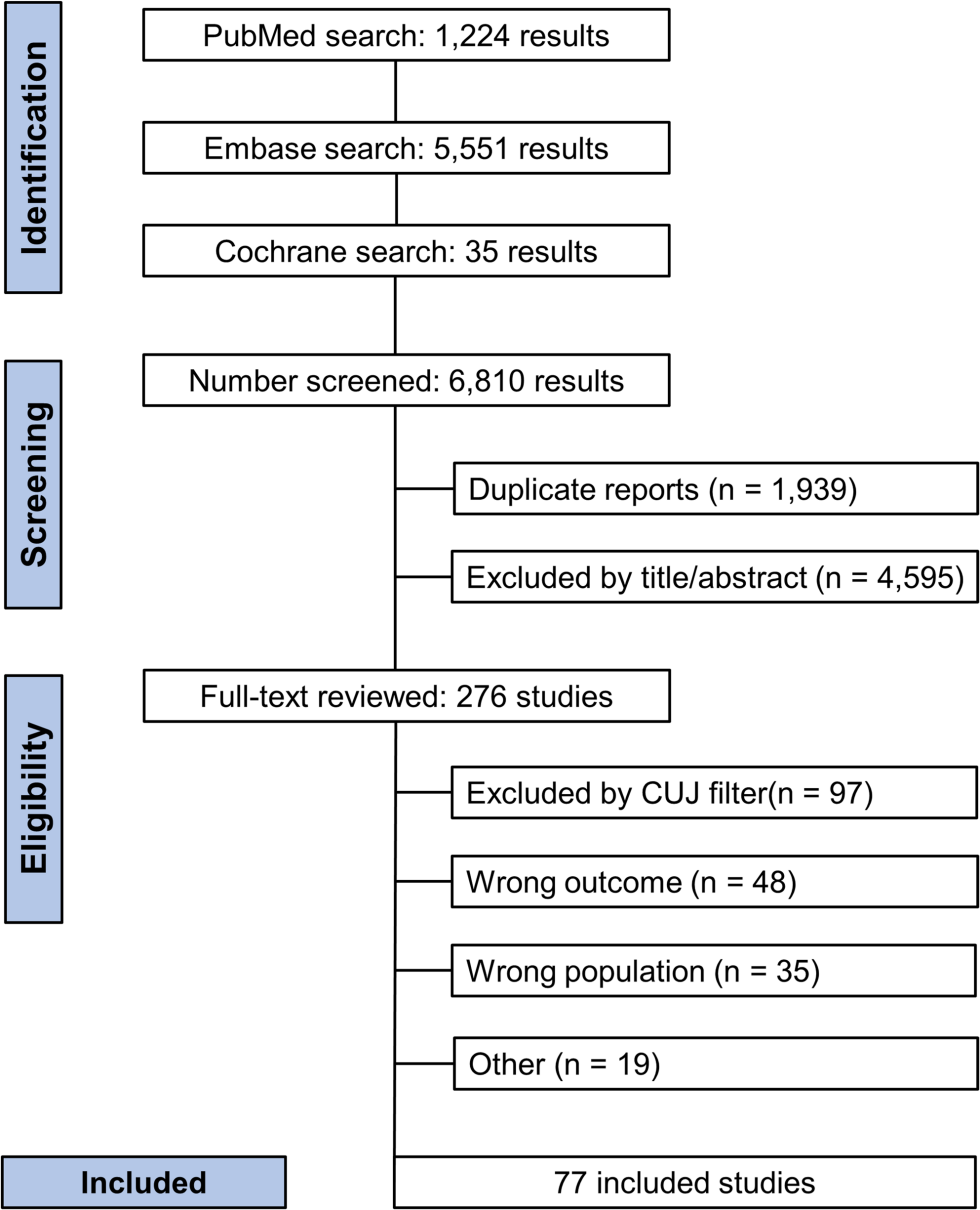
PRISMA flow diagram of study screening and selection

These trials enrolled 144,548 critically ill adult patients. Sample sizes ranged from 106 to 26,828 participants. The mean age of participants across studies ranged from 37 to 75 years, and the proportion of female participants ranged from 18.8% to 58.2%.The PRISMA flow diagram summarizing study selection is presented in Fig. 1.

### Reporting and inclusion of patients with cancer

Among the 77 included trials, 24 studies (31.1%) explicitly reported inclusion of patients with cancer, whereas 9 studies (11.6%) explicitly excluded patients with cancer according to their eligibility criteria. In the remaining 44 trials (57.1%), cancer status was not reported in eligibility criteria, baseline characteristics, or supplementary materials.

Among trials reporting prevalence data, the proportion of enrolled patients with cancer ranged from 3% to 33%. Only four trials reported inclusion of 20% or more participants with cancer [16–19]. No included trial reported mortality outcomes stratified according to oncologic status, and none of the trials were specifically designed to evaluate treatment effects in patients with cancer. 

### Distribution across intervention domains

Inter-reviewer agreement for study classification was excellent (Cohen’s κ = 0.94).

In pharmacologic and immunomodulatory therapies domain (*n* = 20), which included studies on corticosteroids, antimicrobials, immunomodulators, and vitamin therapies, cancer patient inclusion was reported in 40% of studies. However, the reported prevalence was typically low, ranging from 2.5% to 26%, and oncologic status was often omitted or ambiguously categorized as part of broader exclusions.

The hemodynamic and resuscitation strategies domain (*n* = 19) encompassed studies focused on fluids, vasopressors, red blood cell transfusion thresholds, and perfusion targets. Despite the central role of hemodynamic support in ICU management, only four studies (21%) included patients with cancer [20–23].

Mechanical ventilation and oxygenation studies (*n* = 16) included studies on ARDS management, oxygenation strategies, and prone positioning. This domain had a moderate inclusion rate (*n* = 6, 37.5%), though prevalence data were frequently missing. Some studies, such as those by Guerin and Girardis, documented inclusion of patients with cancer, yet none performed stratified analyses [17, 24, 25].

In the renal and metabolic support domain (*n* = 11), which included studies investigating continuous renal replacement therapy (CRRT), insulin protocols, and nutritional strategies, 36.3% of studies explicitly included oncologic patients, marking the highest inclusion rate among all domains. Although prevalence data were inconsistently reported, several studies—such as Schefold et al. and Van den Berghe et al. —included patients with both hematologic and solid tumors [19, 26]. Nonetheless, as in other domains, no dedicated subgroup analyses were performed, and outcomes for patients with cancer remained underreported (Table 1).

**Table 1:** Individual study characteristics

Author	Year	Nº of patients	Female, *n* (%)	Age, years (mean)	ICU population	Type of intervention
Annane	2002	299	99 (33.1)	61	Septic shock	Pharmacologic & Immunomodulatory Therapies
Annane	2007	330	128 (28)	63	Septic shock	Hemodynamic & Resuscitation
Annane	2010	509	179 (35.2)	64	Septic shock	Pharmacologic & Immunomodulatory Therapies
Annane	2013	2,857	1,075 (37.6)	62.8	Hypovolemic shock	Hemodynamic & Resuscitation
Annane	2018	1,241	414 (33.4)	66	Septic shock	Pharmacologic & Immunomodulatory Therapies
Arabi	2008	523	132 (25.2)	52.4	Medical and surgical	Renal & Metabolic Support
Barrot	2020	201	72 (35.8)	63.3	ARDS	Mechanical Ventilation & Oxygenation
Bellomo	2009	1,465	519 (35.4)	64.5	AKI	Renal & Metabolic Support
Bloss	2017	4,183	1,572 (37.6)	70	Septic shock	General ICU Management & Miscellaneous
Brower	2004	549	247 (45)	51.1	ARDS	Mechanical Ventilation & Oxygenation
Busund	2002	106	46 (43.4)	44.4	Septic shock	Pharmacologic & Immunomodulatory Therapies
Caironi	2014	1,819	717 (39.6)	68.2	Septic shock	Hemodynamic & Resuscitation
Casey	2025	2,365	990 (41.8)	60	Mixed population	Pharmacologic & Immunomodulatory Therapies
Cavalcanti	2017	1,010	379 (37.5)	50.9	ARDS	Mechanical Ventilation & Oxygenation
Chastre	2003	401	112 (27.9)	60.5	VAP	Pharmacologic & Immunomodulatory Therapies
Constantin	2019	400	124 (31)	62	ARDS	Mechanical Ventilation & Oxygenation
Dale	2021	3,260	1,248 (38.3)	59.8	General ICU	General ICU Management & Miscellaneous
Dark	2025	2,748	1,091 (39.7)	60.2	Sepsis	Pharmacologic & Immunomodulatory Therapies
De Backer	2010	1,679	723 (43.1)	66.3	Shock	Hemodynamic & Resuscitation
Dulhunty	2024	7,031	2,423 (34.4)	59.5	Sepsis	Pharmacologic & Immunomodulatory Therapies
Ferguson	2013	548	228 (41.6)	54.5	ARDS	Mechanical Ventilation & Oxygenation
Finfer	2004	6,997	2,800 (40)	58.5	Mixed population	Hemodynamic & Resuscitation
Finfer	2009	6,030	2,207 (36.6)	60.2	Mixed population	Renal & Metabolic Support
Girardis	2016	434	188 (43.3)	63.5	Mixed population	Mechanical Ventilation & Oxygenation
Guerin	2004	791	198 (25)	62.2	ARF	Mechanical Ventilation & Oxygenation
Guerin	2013	466	148 (31.8)	59	ARDS	Mechanical Ventilation & Oxygenation
He	2021	117	40 (34.18)	60.3	ARDS	Mechanical Ventilation & Oxygenation
Hernandez	2019	424	226 (53.3)	63	Septic shock	Hemodynamic & Resuscitation
Hernandez	2025	1,467	636 (43.3)	66	Septic shock	Hemodynamic & Resuscitation
Holst	2014	998	467 (46.8)	67	Septic shock	Hemodynamic & Resuscitation
Jansen	2010	348	127 (36.5)	62	Mixed population	Hemodynamic & Resuscitation
Jung	2025	627	248 (39.5)	67	AKI + Severe metabolic acidemia	Renal & Metabolic Support
Kalfon	2014	2,648	942 (35.6)	61.5	Mixed population	Renal & Metabolic Support
Karnad	2014	114	26 (22.8)	37.1	Sepsis	Pharmacologic & Immunomodulatory Therapies
Krag	2018	3,298	1,185 (36)	67	Mixed population	Pharmacologic & Immunomodulatory Therapies
Lamontagne	2020	2,455	1,067 (43.5)	75.3	Vasodilatory shock	Hemodynamic & Resuscitation
Le May	2021	367	69 (18.8)	61.3	Comatose OHCA	General ICU Management & Miscellaneous
Lopez	2004	797	305 (38.3)	64	Septic shock	Pharmacologic & Immunomodulatory Therapies
Lyu	2022	426	141 (33.1)	69.5	Septic shock	Pharmacologic & Immunomodulatory Therapies
Matchett	2022	791	303 (38.3)	55.6	Mixed population	General ICU Management & Miscellaneous
McNamee	2021	412	143 (34.7)	60.3	ARDS	Mechanical Ventilation & Oxygenation
Meade	2008	983	394 (40.1)	55.7	ARDS	Mechanical Ventilation & Oxygenation
Mehta	2001	166	40 (24.1)	55.4	ARF	Renal & Metabolic Support
Mercat	2008	767	251 (32.7)	59.9	ARDS	Mechanical Ventilation & Oxygenation
Meyhoff	2022	1,531	627 (40.9)	70.5	Septic shock	Hemodynamic & Resuscitation
Mohamed	2023	106	31 (29.2)	49.2	Septic shock	Pharmacologic & Immunomodulatory Therapies
Muller	2025	1,006	324 (32.2)	66	Shock	General ICU Management & Miscellaneous
Myburgh	2022	5,982	2,202 (36.8)	58.3	Mechanical ventilation	General ICU Management & Miscellaneous
Olsen	2020	700	273 (39)	71	Mechanical ventilation	General ICU Management & Miscellaneous
Papazian	2013	284	71 (25)	59	VAP	Pharmacologic & Immunomodulatory Therapies
Park	2016	212	74 (34.9)	62.1	Sepsis + AKI	Renal & Metabolic Support
Payen	2015	232	98 (42.2)	71.7	Septic shock	General ICU Management & Miscellaneous
Petilla	2025	194	113 (58.2)	62	Septic shock	Hemodynamic & Resuscitation
Rhodes	2002	201	NA	65.6	Mixed population	Hemodynamic & Resuscitation
Richard	2003	676	224 (33.1)	62.7	ARDS	Hemodynamic & Resuscitation
Richard	2024	699	218 (31.2)	62	ARDS	Mechanical Ventilation & Oxygenation
Rivers	2001	263	130 (49.4)	65.7	Septic shock	Hemodynamic & Resuscitation
Schefold	2014	250	94 (37.6)	61.55	AKI	Renal & Metabolic Support
Schjørring	2021	2,910	1,039 (35.7)	70	ARF	Mechanical Ventilation & Oxygenation
Semler	2018	15,802	6,705 (42.4)	58	Mixed population	Hemodynamic & Resuscitation
Shapiro	2023	1,563	737 (47.2)	59.5	Sepsis	Hemodynamic & Resuscitation
Smith	2012	326	114 (35)	55	ARDS	General ICU Management & Miscellaneous
Sprung	2008	499	167 (33.5)	63	Septic shock	Pharmacologic & Immunomodulatory Therapies
Stephens	2008	164	75 (45.7)	49.9	Septic shock	Pharmacologic & Immunomodulatory Therapies
Taccone	2009	342	98 (28.7)	60	ARDS	Mechanical Ventilation & Oxygenation
Tongyoo	2016	197	96 (48.7)	64.4	Sepsis + ARDS	Pharmacologic & Immunomodulatory Therapies
Van den Berghe	2001	1,548	447 (28.9)	62.8	Mixed population	Renal & Metabolic Support
Van der Wal	2023	664	229 (34.5)	67	Mechanical ventilation	Mechanical Ventilation & Oxygenation
Wacker	2022	124	61 (49.2)	70.9	Septic shock	Pharmacologic & Immunomodulatory Therapies
Warren	2001	2,314	891 (38.5)	57.5	Sepsis	Pharmacologic & Immunomodulatory Therapies
Wheeler	2006	1,000	467 (46.7)	49.7	ARDS	Hemodynamic & Resuscitation
Wolfrum	2022	238	86 (36.1)	72.6	IHCA	General ICU Management & Miscellaneous
Young	2013	899	372 (41.4)	63.9	Mechanical ventilation	General ICU Management & Miscellaneous
Young	2020	26,828	9,691 (36.1)	58.4	Mechanical ventilation	Pharmacologic & Immunomodulatory Therapies
Zampieri	2021	10,520	4,655 (44.2)	61.1	Mixed population	Hemodynamic & Resuscitation
Zarbock	2016	231	85 (36.8)	67	AKI	Renal & Metabolic Support
Zarbock	2020	596	183 (30.7)	67.5	AKI	Renal & Metabolic Support

*AKI* acute kidney injury, *ARDS* acute respiratory distress syndrome, *ARF* acute respiratory failure, *IHCA* in-hospital cardiac arrest, *OHCA* out-of-hospital cardiac arrest, *VAP* ventilator-associated pneumonia

**Fig. 2:**
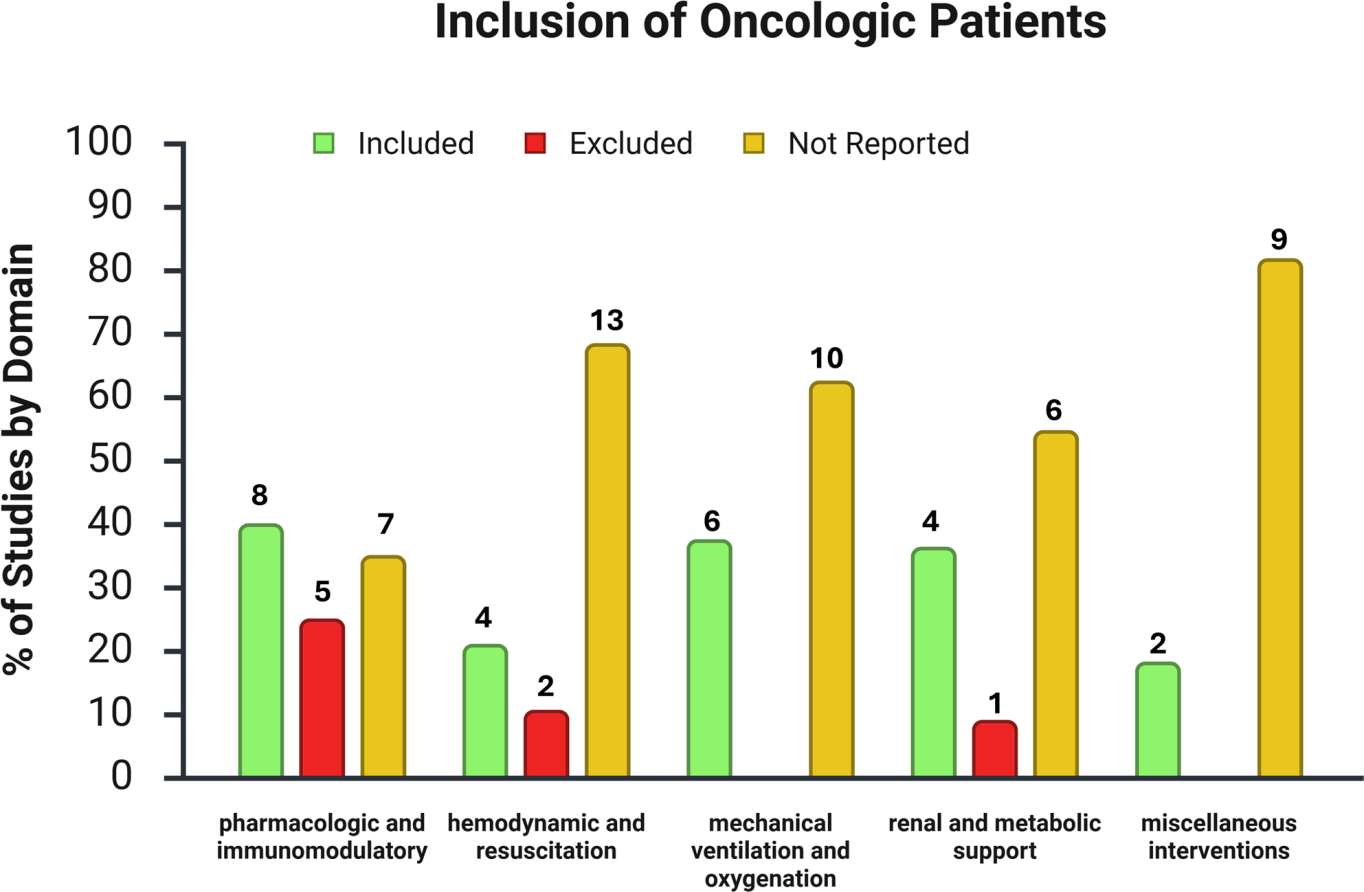
Inclusion of oncologic patients across randomized controlled trials by domain. Bars show the percentage of trials within each domain that explicitly included (green), excluded (red), or did not report (gold) oncologic status (percentages sum to 100% per domain)

Finally, in the general ICU management and miscellaneous domain (*n* = 11), which encompassed studies on sedation protocols, temperature control, bundled care interventions, and organizational strategies only two studies included patients with cancer [18, 27], showing the lowest level of inclusion. A complete graphical dataset of all domains is shown on Fig. 2.

### Temporal patterns in reporting of cancer status

A temporal analysis comparing earlier trials (2000–2019) with more recent studies (2020–2025) showed modest changes in reporting practices (Table 2). Among trials published between 2000 and 2019, 17 studies (28.8%) explicitly included patients with cancer, 9 (15.3%) explicitly excluded them, and 33 (55.9%) did not report cancer status. In contrast, among trials published between 2020 and 2025, 11 studies (40.7%) reported inclusion of patients with cancer, none explicitly excluded malignancy, and 16 (59.3%) did not report cancer status. Although explicit exclusion of patients with cancer was not observed in more recent trials and reporting of inclusion appeared somewhat more frequent, absence of reporting remained common across both periods.

**Table 2:** Comparison of oncology patient inclusion status in ICU mortality-focused randomized trials across study periods (2000–2019 vs 2020–2025)

Inclusion Status	2000–2019	2020–2025
Included	17 (28.8%)	11 (40.7%)
Excluded	9 (15.3%)	0
Not reported	33 (55.9%)	16 (59.3%)

### Risk of bias

Overall, most studies were judged to have a low risk of bias across the majority of domains. The randomization process (Domain 1) and measurement of the outcome (Domain 4) were consistently rated as low risk in nearly all trials. Some studies presented some concerns, most commonly related to deviations from intended interventions (Domain 2) or selection of the reported result (Domain 5), largely reflecting limited reporting of analysis plans or insufficient methodological detail in the published manuscripts. Only one trial was judged to have high risk of bias overall. A complete study-level risk-of-bias assessment across all RoB-2 domains is provided in Supplementary Appendix Figure S1.

## Discussion

This systematic review examined how patients with cancer were reported, explicitly included or excluded, and analytically represented in adult ICU randomized controlled trials that prespecified all-cause mortality as a primary or co-primary outcome. Three main findings emerged. First, cancer status was frequently not reported. Second, explicit inclusion of patients with cancer was limited and explicit exclusion was present in a smaller but still relevant proportion of trials. Third, none of the included trials reported mortality outcomes stratified according to cancer status. Together, these findings suggest that the applicability of mortality-focused ICU trial evidence to patients with cancer is often difficult to assess from published trial reports [8–10].

The main contribution of this review is not simply to show that some ICU trials excluded patients with cancer. Rather, it highlights that non-reporting itself is a major and underrecognized limitation. More than half of included trials did not explicitly describe whether patients with cancer were eligible, enrolled, or represented in baseline characteristics. This lack of reporting is important because it prevents readers from distinguishing between true absence, selective exclusion, and routine inclusion without documentation. In this context, incomplete reporting may be as consequential as explicit exclusion for clinicians trying to judge external validity.

These findings extend previous concerns raised in more limited settings, particularly in sepsis and infection-related trials [8–10]. Prior studies have suggested that malignancy may be restricted through explicit eligibility criteria or through broader constructs such as terminal illness or severe immunosuppression. Our review expands this discussion across a wider range of ICU interventions and focuses specifically on trials designed around mortality endpoints, which are among the studies most likely to influence general ICU practice and guideline development. In addition, the present review examined domain-specific patterns and identified the consistent absence of cancer-specific mortality analyses across all included studies.

The variation observed across intervention domains should be interpreted cautiously. Trials of pharmacologic or immunomodulatory therapies and renal or metabolic support appeared more likely to report inclusion of patients with cancer than trials focused on hemodynamic management or general ICU practices. However, these domain-level differences were not formally modeled and may reflect multiple factors, including differences in trial populations, disease severity, enrollment practices, publication periods, and reporting conventions. In addition, the relatively small number of trials within each domain (ranging from 11 to 20) limits the stability of the observed proportions. Accordingly, these findings are best viewed as descriptive and hypothesis-generating rather than as evidence of systematic domain-specific exclusion.

The absence of oncology-specific outcome analyses was notable. Even in trials in which patients with cancer were explicitly reported in the study population, no trial presented mortality results according to cancer status. This does not imply that meaningful heterogeneity of treatment effect necessarily exists, nor was the present review designed to test effect modification. However, the lack of stratified reporting limits the ability of readers to assess whether trial findings are equally applicable to patients with cancer, particularly given the clinical heterogeneity of this population and the increasing use of ICU support in modern oncology care [4–7, 28–36].

These findings are relevant to anesthesiology and perioperative critical care as well as to general intensive care medicine. In many health systems, anesthesiologists play a central role in ICU management, postoperative rescue pathways, airway and ventilatory support, hemodynamic stabilization, and perioperative care of high-risk surgical patients [37, 38]. Patients with cancer are frequently exposed to major oncologic surgery, treatment-related organ dysfunction, immunologic vulnerability, and unplanned ICU admission. As a result, uncertainty regarding whether such patients are adequately represented or transparently reported in ICU randomized trials has direct implications for perioperative risk stratification, postoperative critical care, and the interpretation of evidence used in anesthesiology-led critical care practice.

The present findings should not be interpreted as definitive proof of formal underrepresentation relative to the true epidemiology of ICU admissions. Our study was not designed to compare trial enrollment directly with expected cancer prevalence across all ICU settings, case mixes, and regions. Reported prevalence of cancer among ICU admissions varies substantially according to center type, malignancy profile, and admission policy [28–31]. Therefore, the strongest conclusion supported by our data is that cancer status is frequently underreported and that explicit inclusion is limited in published mortality-focused ICU randomized trials. Underrepresentation is plausible, but it cannot be established definitively from the present data alone.

Several explanations may contribute to the observed pattern, although these mechanisms were not directly evaluated in our review. Trialists may be concerned about prognostic heterogeneity, cancer-related competing risks, limited life expectancy in selected subgroups, or practical challenges in enrollment and consent. In addition, some reports may have enrolled patients with cancer without explicitly documenting this in the published manuscript. For these reasons, caution is warranted when interpreting non-reporting as exclusion. At the same time, from the standpoint of external validity, lack of transparent reporting remains problematic regardless of the underlying reason [8–10].

Our findings support several practical recommendations for future ICU trials. At a minimum, trial reports should state whether patients with active malignancy were eligible and whether they were represented in the enrolled population. Baseline tables should distinguish active cancer from remote cancer history whenever feasible. When there is a clear biological or clinical rationale, and when sample size permits, prespecified subgroup analyses according to cancer status may be informative. However, such analyses should not be performed indiscriminately, because interaction testing requires adequate power and cancer is itself a heterogeneous clinical construct. A pragmatic reporting standard may therefore be more broadly achievable than universal subgroup testing.

This review has limitations. First, it relied on published reports rather than individual participant data, and some trials may have enrolled patients with cancer without explicitly documenting this in the main article or supplementary appendix. Second, the restriction to English-language trials published in Core Clinical Journals was prespecified to capture influential ICU randomized trials, but it may have excluded relevant studies from subspecialty journals and therefore limits generalizability [15]. In addition, we did not perform a preliminary unrestricted search prior to applying the Core Clinical Journals filter. As a result, we are unable to estimate the number of additional trials that may have been identified without this restriction. This limits our ability to quantify the potential impact of the filter on study selection. Third, classification of cancer status depended on the terminology used by trial authors, and although prespecified decision rules were applied, some degree of residual misclassification is possible. Fourth, the domain-based analyses were descriptive and were not intended to test causal explanations for differences in reporting patterns across intervention categories. Finally, because this review did not include a direct comparison between trial representation and expected cancer prevalence in ICU populations, it cannot quantify underrepresentation in a formal epidemiologic sense.

## Conclusion

In this systematic review of adult ICU randomized controlled trials powered for mortality outcomes, cancer status was frequently not reported, explicit inclusion of patients with cancer was limited, and oncology-specific outcome analyses were absent. These findings make it difficult to determine how well the results of influential ICU trials apply to patients with cancer, a population that represents a growing proportion of critically ill patients in many settings.

More consistent reporting of cancer status, clearer description of eligibility criteria related to malignancy, and selective use of prespecified oncology-focused analyses when clinically justified may improve the interpretability and applicability of future ICU randomized trials.

## Supplementary Information

Supplementary Material 1.

## Data Availability

The data that support the findings of this study are available from the corresponding author (RHM) upon reasonable request.

## Abbreviations

AKI: Acute Kidney Injury
ARDS: Acute Respiratory Distress Syndrome
CRRT: Continuous Renal Replacement Therapy
ICMJE: International Committee of Medical Journal Editors
ICU / ICUs: Intensive Care Unit(s)
MeSH: Medical Subject Headings
PRISMA: Preferred Reporting Items for Systematic Reviews and Meta—Analyses
PROSPERO: International Prospective Register of Systematic Reviews
RCT / RCTs: Randomized Controlled Trial(s)
