# Supplementary material for: Representation and reporting of patients with cancer in mortality-focused adult ICU randomized trials: a systematic review
Source: BMC Anesthesiol. 2026 Apr 20;26:346. doi: 10.1186/s12871-026-03840-w (PMC13244898; doi:10.1186/s12871-026-03840-w)
Supplement: Supplementary file 1 — Supplementary Material 1. [file 12871_2026_3840_MOESM1_ESM.docx]

**Supplementary Material**

Underrepresentation and Mortality Outcomes of Oncology Patients in Randomized Clinical Trials in Intensive Care Medicine: A Systematic Review

Rafael Hortêncio Melo1,2, Luciana Gioli-Pereira1,2, Mariana Resende Bustamante1, Gustavo Potratz Gonçalves1, Valéria Urresti Orias1, Amanda Pascoal Valle Felício1,3, Thiago Domingos Corrêa1,3, Rogério Da Hora Passos3

1Hospital Municipal Gilson de Cássia Marques de Carvalho; Hospital Israelita Albert Einstein, São Paulo,SP, Brazil

2Faculdade Israelita de Ciências da Saúde Albert Einstein, Hospital Israelita Albert Einstein, São Paulo,SP, Brazil

3Hospital Israelita Albert Einstein, São Paulo, SP, Brazil

**Contents**

[**Search Strategy** 2](#_Toc222773133)

[**Risk of Bias Evaluation – RoB 2** 3](#_Toc222773134)

[**Supplementary Figure 1 – Risk of Bias** 27](#_Toc222773135)

[**Supplementary Table 1 – Domain comparison 2000-2019 vs 2020-2025** 28](#_Toc222773136)

[**Table 1 – Complete Version** 30](#_Toc222773137)

[**PRISMA CHECKLIST** 36](#_Toc222773138)

# **Search Strategy**

| Database | Complete Query |
| --- | --- |
| PubMed | ("Intensive Care Units"[MeSH] OR ''intensive care unit'' OR "Critical Illness"[MeSH] OR "critically ill patients" OR "ICU" OR "sepsis" OR "septic shock" OR "multiple organ failure" OR ''shock'' OR ''shock''[MeSH] OR "acute respiratory distress syndrome" OR "ARDS") AND ("Mortality"[MeSH] OR "hospital mortality" OR "ICU mortality" OR "all-cause mortality" OR "30-day mortality" OR "90-day mortality" OR "survival rate") AND ("Outcome"[Title/Abstract] OR "Clinical outcomes"[Title/Abstract] OR "Effect"[Title/Abstract]) AND ("Randomized Controlled Trial"[Publication Type]) AND (Humans[MeSH] AND English[lang]) AND ("Adult"[MeSH] OR "Adults"[Title/Abstract]) NOT ("Pediatric"[Title/Abstract] OR "Child"[MeSH] OR "Neonatal"[Title/Abstract]) |
| Cochrane Library | (Intensive Care Units OR "intensive care unit" OR Critical Illness OR "critically ill patients" OR ICU OR Sepsis OR "septic shock" OR "multiple organ failure" OR Shock OR "acute respiratory distress syndrome" OR ARDS) AND (Mortality OR "hospital mortality" OR "ICU mortality" OR "all-cause mortality" OR "30-day mortality" OR "90-day mortality" OR "survival") AND (Outcome OR "clinical outcomes" OR Effect OR Results OR Findings) AND (Trial OR "randomized" OR "randomised" OR "controlled trial" OR "RCT") AND Humans AND English NOT (Pediatric OR Child OR Neonatal) |
| EMBASE | ('intensive care unit'/exp OR 'intensive care unit' OR 'critical illness'/exp OR 'critically ill patients' OR 'ICU' OR 'sepsis'/exp OR 'sepsis' OR 'septic shock'/exp OR 'septic shock' OR 'multiple organ failure'/exp OR 'multiple organ failure' OR 'shock'/exp OR 'shock' OR 'acute respiratory distress syndrome'/exp OR 'ARDS') AND ('mortality'/exp OR 'hospital mortality' OR 'ICU mortality' OR 'all-cause mortality' OR '30-day mortality' OR '90-day mortality' OR 'survival rate') AND ('outcome':ti,ab OR 'clinical outcomes':ti,ab OR 'effect':ti,ab) AND ('randomized controlled trial'/exp) AND ('human'/exp) AND ('adult'/exp) AND [english]/lim NOT ('pediatrics'/exp OR 'child'/exp OR 'neonatal'/exp)AND ('adult'/exp) AND [english]/lim NOT ('pediatrics'/exp OR 'child'/exp OR 'neonatal'/exp) |

# **Risk of Bias Evaluation – RoB 2**

| Annane 2002 | D1 – Randomization process: Low risk  Centralized computer-generated randomization  Stratified by center, concealed allocation  Baseline characteristics balanced  D2 – Deviations from intended interventions: Low risk  Double-blind (patients, clinicians, pharmacists)  ITT analysis performed  No relevant protocol deviations affecting primary outcome  D3 – Missing outcome data: Low risk  No loss to follow-up  One consent withdrawal (excluded appropriately)  Complete 28-day outcome data  D4 – Measurement of the outcome: Low risk  Mortality is objective  Blinded assessment  No risk of measurement bias  D5 – Selection of the reported result: Low risk  Primary endpoint predefined  Appropriate statistical methods  No evidence of selective reporting  Overall risk of bias: LOW |
| --- | --- |
| Annane 2007 | D1 – Randomization process: Low risk  Central computerized randomization  Stratified by center  Adequate concealment  D2 – Deviations from intended interventions: Low risk  Double-blind  ITT analysis  Minimal protocol deviations  D3 – Missing outcome data: Low risk  No loss to follow-up  One consent withdrawal after day 3  Complete primary outcome data  D4 – Measurement of the outcome: Low risk  Mortality is objective  Blinded assessment  D5 – Selection of the reported result: Low risk  Trial registered  Prespecified primary endpoint  Consistent reporting  Overall risk of bias: LOW |
| Annane 2010 | D1 – Randomization process: Low risk  Central web-based randomization  Stratified by center  Block randomization  Groups balanced  D2 – Deviations from intended interventions: Some concerns  Open-label insulin strategy  Clinicians not blinded  Mortality unlikely affected, but performance bias possible  D3 – Missing outcome data: Low risk  Complete primary outcome data  No meaningful attrition  D4 – Measurement of the outcome: Low risk  Mortality objective  No differential assessment  D5 – Selection of the reported result: Low risk  Registered trial  Prespecified primary endpoint  Overall risk of bias: LOW (with minor concerns in D2) |
| Annane 2013 | D1 – Randomization process: Low risk  Computer-generated randomization  Stratified by center and case-mix  Allocation concealment via sealed envelopes  D2 – Deviations from intended interventions: Some concerns  Open-label fluid assignment  Clinicians aware of group allocation  Co-interventions possible (though mortality objective)  D3 – Missing outcome data: Low risk  Large sample  Complete 28-day follow-up  D4 – Measurement of the outcome: Low risk  Mortality objective  Blinded outcome assessors  D5 – Selection of the reported result: Low risk  Registered trial  Prespecified 28-day mortality  Overall risk of bias: LOW (with some concerns in D2) |
| Annane 2018 | D1 – Randomization process: Low risk  Central randomization  Block randomization  2×2 factorial design  Proper concealment  D2 – Deviations from intended interventions: Low risk  Double-blind  Matching placebos  ITT analysis  D3 – Missing outcome data: Low risk  Complete 90-day follow-up  Minimal attrition  D4 – Measurement of the outcome: Low risk  Mortality objective  Blinded outcome assessment  D5 – Selection of the reported result: Low risk  Protocol and SAP published  Prespecified primary outcome  Transparent reporting  Overall risk of bias: LOW |
| Arabi 2008 | D1 – Randomization process: Low risk  Computer-generated randomization  Allocation concealment described  Baseline characteristics balanced  D2 – Deviations from intended interventions: Low risk  Double-blind, placebo-controlled  Clinicians and patients blinded  ITT analysis performed  D3 – Missing outcome data: Low risk  Minimal attrition  Complete 28-day mortality data  D4 – Measurement of the outcome: Low risk  Mortality objective  Blinding maintained  D5 – Selection of the reported result: Low risk  Prespecified primary endpoint  No evidence of selective reporting  Overall risk of bias: LOW |
| Barrot 2020 | D1 – Randomization process: Low risk  Computer-generated block randomization  Stratified by center and severity  Adequate allocation process  D2 – Deviations from intended interventions: Some concerns  Open-label design  Oxygen targets visible to clinicians  Mortality objective, but co-interventions possible  D3 – Missing outcome data: Low risk  Only 4 ineligible excluded  Complete 28-day mortality data  D4 – Measurement of the outcome: Low risk  Mortality objective  No differential outcome assessment  D5 – Selection of the reported result: Low risk  Registered (NCT02713451)  Primary endpoint prespecified  Overall risk of bias: LOW (with some concerns in D2) |
| Bellomo 2009 | D1 – Randomization process: Low risk  Centralized randomization  Large multicenter design  Balanced baseline characteristics  D2 – Deviations from intended interventions: Some concerns  Open-label  CRRT dose not blinded  However, mortality unlikely influenced  D3 – Missing outcome data: Low risk  Primary outcome available for 97%  Very low attrition  ITT analysis  D4 – Measurement of the outcome: Low risk  Mortality objective  No detection bias  D5 – Selection of the reported result: Low risk  Registered (NCT00221013)  Statistical plan predefined  Overall risk of bias: LOW (minor concerns in D2) |
| Bloos 2017 | D1 – Randomization process: Some concerns  Cluster randomization  Baseline imbalance (higher illness severity in intervention group)  Stratification performed, but imbalance remained  D2 – Deviations from intended interventions: High risk  Open-label  Cluster-level intervention  Potential contamination and secular trends  Recruitment imbalance  D3 – Missing outcome data: Some concerns  155 patients missing 28-day mortality  Missingness not fully detailed  D4 – Measurement of the outcome: Low risk  Mortality objective  D5 – Selection of the reported result: Low risk  Registered (NCT01187134)  Primary outcome prespecified  Overall risk of bias: HIGH  (Driven mainly by cluster design complexities and deviations) |
| Brower 2004 | D1 – Randomization process: Low risk  Centralized randomization  Stratified by hospital  Concealment adequate  D2 – Deviations from intended interventions: Some concerns  Open-label ventilation strategy  Protocol modification mid-trial (PEEP adjustment)  Steering committee blinded to outcomes when change made  D3 – Missing outcome data: Low risk  Very large multicenter RCT  Minimal loss to follow-up  D4 – Measurement of the outcome: Low risk  Mortality objective  D5 – Selection of the reported result: Low risk  Prespecified primary endpoint  No evidence of selective reporting  Overall risk of bias: LOW (with minor concerns in D2) |
| Caironi 2014 | D1: Bias arising from the randomization process  Centralized computer-generated randomization, stratified by site and timing of sepsis onset. Baseline characteristics were balanced.  Judgment: Low risk  D2: Bias due to deviations from intended interventions  Open-label design. However, mortality is an objective outcome and analysis was by intention-to-treat. No clear evidence of differential cointerventions affecting mortality.  Judgment: Some concerns  D3: Bias due to missing outcome data  Vital status at 28 days was nearly complete. No evidence of differential loss.  Judgment: Low risk  D4: Bias in measurement of the outcome  All-cause mortality is objective and unlikely to be influenced by lack of blinding.  Judgment: Low risk  D5: Bias in selection of the reported result  Primary outcome was clearly pre-specified and protocol was available.  Judgment: Low risk  Overall: Low risk |
| Casey 2025 | D1: Bias arising from the randomization process  Permuted blocks with stratification by site. Allocation concealed using opaque sequential envelopes. Groups well balanced.  Judgment: Low risk  D2: Bias due to deviations from intended interventions  Unblinded trial. Clinicians were aware of assignment, and cointerventions (vasopressors, corticosteroids) were not protocolized. However, ITT analysis was used and outcome is objective.  Judgment: Some concerns  D3: Bias due to missing outcome data  Minimal loss to follow-up. Vital status reliably obtained.  Judgment: Low risk  D4: Bias in measurement of the outcome  Mortality is objective and unlikely to be influenced by knowledge of intervention.  Judgment: Low risk  D5: Bias in selection of the reported result  Protocol and statistical analysis plan were published prior to completion. Primary outcome consistent with registration.  Judgment: Low risk  Overall: Low risk |
| Cavalcanti 2017 | D1: Bias arising from the randomization process  Central web-based randomization with stratification by site and key variables. Baseline characteristics comparable.  Judgment: Low risk  D2: Bias due to deviations from intended interventions  Open-label design. Complex intervention. Protocol modification during the trial (change in recruitment maneuver parameters after safety events), which may introduce heterogeneity. ITT analysis used.  Judgment: Some concerns  D3: Bias due to missing outcome data  Minimal missing data for 28-day mortality.  Judgment: Low risk  D4: Bias in measurement of the outcome  Mortality is objective and unlikely to be affected by lack of blinding.  Judgment: Low risk  D5: Bias in selection of the reported result  Primary outcome pre-specified in protocol and trial registration.  Judgment: Low risk  Overall: Low risk |
| Chastre 2003 | D1: Bias arising from the randomization process  Central telephone randomization, stratified by center in blocks. Baseline balance reported.  Judgment: Low risk  D2: Bias due to deviations from intended interventions  Double-blind until day 8, then open-label. Intervention concerned antibiotic duration. ITT analysis performed. Mortality objective.  Judgment: Some concerns  D3: Bias due to missing outcome data  Very limited missing outcome data.  Judgment: Low risk  D4: Bias in measurement of the outcome  Mortality is objective and unlikely to be influenced by lack of blinding.  Judgment: Low risk  D5: Bias in selection of the reported result  Primary outcomes pre-defined and consistently reported.  Judgment: Low risk  Overall: Low risk |
| Constantin 2019 | D1: Bias arising from the randomization process  Centralized web-based randomization with concealed allocation. Stratification by site. Baseline characteristics well balanced between groups.  Judgment: Low risk  D2: Bias due to deviations from intended interventions  Open-label design (neuromuscular blockade vs usual care with lighter sedation). Co-interventions (sedation depth, ventilatory management) could differ between groups. However, the primary outcome is mortality and ITT analysis was performed. Deviations unlikely to bias mortality materially.  Judgment: Some concerns  D3: Bias due to missing outcome data  Vital status at 90 days was available for nearly all randomized patients. No evidence of differential loss to follow-up.  Judgment: Low risk  D4: Bias in measurement of the outcome  All-cause mortality at 90 days is objective and not subject to measurement bias.  Judgment: Low risk  D5: Bias in selection of the reported result  Trial registered (ClinicalTrials.gov). Protocol and statistical analysis plan published before completion. Primary outcome pre-specified and reported accordingly.  Judgment: Low risk  Overall: Low risk |
| Dale 2021 | D1: Bias arising from the randomization process  Stepped wedge cluster-randomized design with computer-generated allocation sequence. Randomization at cluster (ICU) level. No major baseline imbalances reported.  Judgment: Low risk  D2: Bias due to deviations from intended interventions  Open-label cluster trial. Intervention involved de-adoption and implementation of a bundle. Deviations at individual level unlikely to affect mortality materially, but cluster-level implementation variability possible.  Judgment: Some concerns  D3: Bias due to missing outcome data  ICU mortality obtained from registry data (iCORE). Vital status in ICU expected to be complete. No evidence of differential missingness.  Judgment: Low risk  D4: Bias in measurement of the outcome  ICU mortality is objective and unlikely to be influenced by lack of blinding.  Judgment: Low risk  D5: Bias in selection of the reported result  Trial registered (NCT03382730). Primary outcome pre-specified. Reporting consistent with protocol.  Judgment: Low risk  Overall: Low risk |
| Dark 2024 | D1: Bias arising from the randomization process  Centralized web-based randomization using minimization with stratification by site and sepsis severity. Allocation concealed. Baseline characteristics balanced.  Judgment: Low risk  D2: Bias due to deviations from intended interventions  Intervention-concealed design for biomarker advice; clinicians blinded to allocation in standard care group via delayed advice system. Mortality unlikely to be influenced by protocol deviations. ITT analysis used.  Judgment: Low risk  D3: Bias due to missing outcome data  Some withdrawals and missing data, but numbers small relative to total sample. No evidence of differential loss affecting mortality.  Judgment: Low risk  D4: Bias in measurement of the outcome  28-day all-cause mortality is objective and not subject to measurement bias.  Judgment: Low risk  D5: Bias in selection of the reported result  Protocol and statistical analysis plan published. Co-primary outcomes clearly defined a priori. Reporting consistent.  Judgment: Low risk  Overall: Low risk |
| De Backer 2010 | D1: Bias arising from the randomization process  Computer-generated permuted blocks stratified by ICU. Allocation via sealed opaque envelopes. Baseline characteristics similar.  Judgment: Low risk  D2: Bias due to deviations from intended interventions  Double-blind administration of study drug. Rescue vasopressors allowed per protocol. ITT analysis performed.  Judgment: Low risk  D3: Bias due to missing outcome data  28-day mortality available for nearly all randomized patients. No evidence of differential loss.  Judgment: Low risk  D4: Bias in measurement of the outcome  Mortality is objective and unlikely to be influenced by treatment knowledge.  Judgment: Low risk  D5: Bias in selection of the reported result  Registered trial (NCT00314704). Primary outcome clearly pre-specified. Reporting consistent with protocol.  Judgment: Low risk  Overall: Low risk |
| Dulhunty 2024 | D1: Bias arising from the randomization process  Central web-based minimization algorithm with stratification by site. Baseline characteristics well balanced.  Judgment: Low risk  D2: Bias due to deviations from intended interventions  Open-label design. However, primary outcome is mortality at 90 days and unlikely to be influenced by lack of blinding. ITT primary analysis used.  Judgment: Low risk  D3: Bias due to missing outcome data  Primary analysis included 97.6% of randomized participants meeting consent requirements. Minimal loss to follow-up for mortality.  Judgment: Low risk  D4: Bias in measurement of the outcome  All-cause mortality is objective and not susceptible to measurement bias.  Judgment: Low risk  D5: Bias in selection of the reported result  Protocol and SAP published before completion. Primary outcome pre-specified and reported accordingly.  Judgment: Low risk  Overall: Low risk |
| Ferguson 2013 | D1: Bias arising from the randomization process  Central web-based randomization with concealed allocation, stratified by center. Baseline characteristics well balanced.  Judgment: Low risk  D2: Bias due to deviations from intended interventions  Open-label ventilation strategy. Higher sedation, neuromuscular blockade, and vasoactive use in HFOV group. However, allocation adhered to protocol and analysis followed intention-to-treat. Mortality objective.  Judgment: Some concerns  D3: Bias due to missing outcome data  In-hospital mortality available for virtually all randomized patients. No evidence of differential attrition.  Judgment: Low risk  D4: Bias in measurement of the outcome  Mortality is objective and unlikely to be influenced by lack of blinding.  Judgment: Low risk  D5: Bias in selection of the reported result  Trial registered; protocol available. Primary outcome clearly pre-specified and reported.  Judgment: Low risk  Overall: Low risk |
| Finfer 2004 | D1: Bias arising from the randomization process  Central randomization using minimization algorithm, stratified by center and trauma status. Allocation concealed. Baseline characteristics similar.  Judgment: Low risk  D2: Bias due to deviations from intended interventions  Double-blind design. Identical bottles and masking procedures. Treatment delivered per protocol. ITT analysis.  Judgment: Low risk  D3: Bias due to missing outcome data  Minimal missing data for 28-day mortality. Analysis performed by intention-to-treat.  Judgment: Low risk  D4: Bias in measurement of the outcome  All-cause mortality objective and not subject to measurement bias.  Judgment: Low risk  D5: Bias in selection of the reported result  Primary outcome pre-specified. Statistical plan defined a priori. Reporting consistent.  Judgment: Low risk  Overall: Low risk |
| Finfer 2009 | D1: Bias arising from the randomization process  Web-based minimization algorithm, stratified by operative status and region. Allocation concealed prior to assignment. Groups well balanced.  Judgment: Low risk  D2: Bias due to deviations from intended interventions  Open-label glucose targets. Clinicians aware of allocation. Protocolized insulin algorithm used in both groups. ITT analysis. Mortality objective.  Judgment: Some concerns  D3: Bias due to missing outcome data  Primary outcome available for nearly all patients (very low missingness). No differential loss.  Judgment: Low risk  D4: Bias in measurement of the outcome  Mortality objective and not susceptible to measurement bias.  Judgment: Low risk  D5: Bias in selection of the reported result  Registered trial (NCT00220987). Pre-specified primary endpoint and statistical analysis plan.  Judgment: Low risk  Overall: Low risk |
| Girardis 2016 | D1: Bias arising from the randomization process  Computer-generated randomization with sealed opaque envelopes. Baseline characteristics comparable.  Judgment: Low risk  D2: Bias due to deviations from intended interventions  Open-label single-center trial. Early termination before planned sample size. Potential risk of performance bias. ITT-based modified analysis.  Judgment: Some concerns  D3: Bias due to missing outcome data  Modified ITT population analyzed (patients with at least one ABG per day). Exclusion of some randomized patients introduces potential bias.  Judgment: Some concerns  D4: Bias in measurement of the outcome  ICU mortality objective.  Judgment: Low risk  D5: Bias in selection of the reported result  Primary outcome clearly defined. Early stopping may influence precision but no evidence of selective reporting.  Judgment: Low risk  Overall: Some concerns |
| Guerin 2004 | D1: Bias arising from the randomization process  Computer-generated randomization with sequentially numbered opaque sealed envelopes. Allocation concealed. Baseline balance reported.  Judgment: Low risk  D2: Bias due to deviations from intended interventions  Unblinded trial. Crossover allowed for severe hypoxemia. Potential deviations in management between groups. ITT principle applied.  Judgment: Some concerns  D3: Bias due to missing outcome data  28-day mortality available for the majority of participants; no indication of significant differential loss.  Judgment: Low risk  D4: Bias in measurement of the outcome  Mortality objective and not influenced by lack of blinding.  Judgment: Low risk  D5: Bias in selection of the reported result  Primary outcome clearly defined in protocol. Reporting consistent.  Judgment: Low risk  Overall: Low risk |
| He 2021 | D1: Bias arising from the randomization process  Computer-generated random block design by an independent operator, with allocation concealed using sequentially numbered opaque sealed envelopes. Baseline characteristics not flagged as meaningfully imbalanced.  Judgment: Low risk  D2: Bias due to deviations from intended interventions  Single-center open-label trial (clinicians aware of group assignment). Co-interventions could differ, although the primary outcome is objective.  Judgment: Some concerns  D3: Bias due to missing outcome data  28-day mortality appears available for essentially all randomized patients; no signal of differential missingness.  Judgment: Low risk  D4: Bias in measurement of the outcome  All-cause mortality is objective.  Judgment: Low risk  D5: Bias in selection of the reported result  Prospectively registered (NCT02361398) with a clearly stated primary outcome.  Judgment: Low risk  Overall: Low risk |
| Hernández 2019 | D1: Bias arising from the randomization process  Central randomization with a computer-generated permuted-block sequence; allocation disclosed only after centralized confirmation. Baseline characteristics comparable.  Judgment: Low risk  D2: Bias due to deviations from intended interventions  Open-label protocolized resuscitation strategies; clinicians unblinded. Outcome is objective and analyzed by intention-to-treat, but co-interventions remain possible.  Judgment: Some concerns  D3: Bias due to missing outcome data  High completeness of follow-up (98% completed the trial) with explicit procedures to minimize loss.  Judgment: Low risk  D4: Bias in measurement of the outcome  Mortality is objective.  Judgment: Low risk  D5: Bias in selection of the reported result  Protocol and statistical analysis plan published; trial registered (NCT03078712).  Judgment: Low risk  Overall: Low risk |
| Hernández 2025 | D1: Bias arising from the randomization process  Central web-based randomization with stratification by center and variable block sizes; allocation concealment ensured.  Judgment: Low risk  D2: Bias due to deviations from intended interventions  Unblinded to clinicians and patients. The intervention is a complex protocol, increasing risk of differential co-interventions that may affect “duration of vital support” and LOS.  Judgment: Some concerns  D3: Bias due to missing outcome data  Some withdrawals and loss to follow-up at 28 days are reported in the flow diagram. Given the composite outcome includes non-mortality components, missingness could matter.  Judgment: Some concerns  D4: Bias in measurement of the outcome  Composite includes duration of life support and LOS, which are susceptible to clinician behavior and discharge practices in an unblinded trial (even if mortality is objective).  Judgment: Some concerns  D5: Bias in selection of the reported result  Trial registered (NCT05057611) with protocol/SAP available; primary outcome prespecified as hierarchical composite.  Judgment: Low risk  Overall: Some concerns |
| Holst 2014 | D1: Bias arising from the randomization process  Central computerized assignment with stratification and permuted blocks of varying sizes; robust allocation concealment. Baseline characteristics similar.  Judgment: Low risk  D2: Bias due to deviations from intended interventions  Open-label transfusion threshold strategy (clinicians unblinded). ITT analysis and objective primary outcome reduce impact, but deviations remain possible.  Judgment: Some concerns  D3: Bias due to missing outcome data  Very high completeness (998/1005 analyzed).  Judgment: Low risk  D4: Bias in measurement of the outcome  90-day mortality objective; outcome assessors/statistician masked as described.  Judgment: Low risk  D5: Bias in selection of the reported result  Protocol and SAP published; registered (NCT01485315).  Judgment: Low risk  Overall: Low risk |
| Jansen 2010 | D1: Bias arising from the randomization process  Randomization described as stratified, but the concealment mechanism is not clearly detailed in the main text excerpt.  Judgment: Some concerns  D2: Bias due to deviations from intended interventions  Open-label design; intervention arm received protocolized lactate-driven treatment while controls had lactate values withheld (except admission). Co-interventions likely differed (fluids/vasodilators), which could influence outcomes.  Judgment: Some concerns  D3: Bias due to missing outcome data  Hospital mortality is typically complete; no clear signal of differential missingness in the report excerpt.  Judgment: Low risk  D4: Bias in measurement of the outcome  Hospital mortality objective.  Judgment: Low risk  D5: Bias in selection of the reported result  Registered (NCT00270673). However, the prominent emphasis on adjusted mortality effects alongside a borderline unadjusted comparison raises some concern about analytic flexibility unless the primary analysis was clearly prespecified as adjusted.  Judgment: Some concerns  Overall: Some concerns |
| Jung 2025 | D1: Bias arising from the randomization process  Centralized, computer-generated randomization with stratification by center, age, and pH strata. Allocation concealment ensured. Baseline characteristics well balanced.  Judgment: Low risk  D2: Bias due to deviations from intended interventions  Open-label design. Intervention (bicarbonate infusion) could influence co-interventions (e.g., timing of KRT), but primary outcome is objective and analysis followed intention-to-treat.  Judgment: Some concerns  D3: Bias due to missing outcome data  Very high completeness of 90-day mortality follow-up; exclusions minimal and balanced.  Judgment: Low risk  D4: Bias in measurement of the outcome  All-cause mortality at 90 days is objective and unlikely to be influenced by lack of blinding.  Judgment: Low risk  D5: Bias in selection of the reported result  Prospectively registered (NCT04010630) with published protocol and SAP. Primary outcome prespecified and reported accordingly.  Judgment: Low risk  Overall: Low risk |
| Kalfon 2014 | D1: Bias arising from the randomization process  Electronic randomization with permuted blocks; allocation concealment described. Baseline characteristics similar across groups.  Judgment: Low risk  D2: Bias due to deviations from intended interventions  Open-label trial. Tight glucose control via CDSS versus heterogeneous conventional protocols. Increased hypoglycemia and protocol-driven care could introduce performance bias.  Judgment: Some concerns  D3: Bias due to missing outcome data  Primary outcome available for the vast majority of randomized patients; missing data balanced.  Judgment: Low risk  D4: Bias in measurement of the outcome  90-day mortality is objective.  Judgment: Low risk  D5: Bias in selection of the reported result  Registered trial (NCT01002482). Primary outcome clearly prespecified and consistently reported.  Judgment: Low risk  Overall: Low risk |
| Karnad 2014 | D1: Bias arising from the randomization process  Computer-generated block randomization with sealed opaque envelopes; allocation concealment adequate.  Judgment: Low risk  D2: Bias due to deviations from intended interventions  Double-blind, placebo-controlled design. However, primary mortality effect emphasized in a modified intention-to-treat analysis rather than the full ITT population.  Judgment: Some concerns  D3: Bias due to missing outcome data  Some attrition and exclusion from the modified ITT analysis; full ITT showed non-significant results. Missingness may be related to intervention exposure.  Judgment: Some concerns  D4: Bias in measurement of the outcome  28-day mortality objective and blinded assessment.  Judgment: Low risk  D5: Bias in selection of the reported result  Primary outcome prespecified, but selective emphasis on modified ITT analysis raises concern regarding analytic flexibility.  Judgment: Some concerns  Overall: Some concerns |
| Krag 2018 | D1: Bias arising from the randomization process  Centralized, computer-generated randomization with stratification by site and cancer status. Robust allocation concealment.  Judgment: Low risk  D2: Bias due to deviations from intended interventions  Double-blind, placebo-controlled design with high adherence. Minimal protocol deviations.  Judgment: Low risk  D3: Bias due to missing outcome data  Primary outcome available for 99.5% of randomized patients.  Judgment: Low risk  D4: Bias in measurement of the outcome  Mortality objective; assessors and analysts blinded.  Judgment: Low risk  D5: Bias in selection of the reported result  Protocol and SAP published a priori; primary outcome reported as planned.  Judgment: Low risk  Overall: Low risk |
| Lamontagne 2020 | D1: Bias arising from the randomization process  Centralized web/telephone randomization with stratification by site and variable block sizes. Allocation concealment adequate.  Judgment: Low risk  D2: Bias due to deviations from intended interventions  Open-label pragmatic design. Clinician-driven co-interventions likely differed between permissive hypotension and usual care. However, ITT analysis performed and outcome objective.  Judgment: Some concerns  D3: Bias due to missing outcome data  Approximately 5% excluded from primary analysis due to withdrawal or refusal of consent. Missingness balanced but non-negligible.  Judgment: Some concerns  D4: Bias in measurement of the outcome  90-day mortality objective.  Judgment: Low risk  D5: Bias in selection of the reported result  Registered trial (ISRCTN10580502) with prespecified primary outcome. Adjusted analyses presented but consistent with protocol.  Judgment: Low risk  Overall: Some concerns |
| Le May 2021 | D1: Bias arising from the randomization process  Computer-generated permuted blocks, stratified by initial rhythm. Allocation concealed with sealed opaque envelopes. Baseline characteristics balanced.  Judgment: Low risk  D2: Bias due to deviations from intended interventions  Double-blind design (physicians, patients, families blinded; only bedside nurses aware). Standardized temperature management protocol.  Judgment: Low risk  D3: Bias due to missing outcome data  367 included in primary analysis out of 389 randomized. Completion rate 99.7% among analyzed patients. Exclusions minimal and unlikely related to outcome.  Judgment: Low risk  D4: Bias in measurement of the outcome  Composite outcome included mortality (objective) and neurologic status assessed using Disability Rating Scale by blinded assessors.  Judgment: Low risk  D5: Bias in selection of the reported result  Registered (NCT02011568). Protocol and SAP available. Primary outcome clearly prespecified.  Judgment: Low risk  Overall: Low risk |
| López 2004 | D1: Bias arising from the randomization process  Multicenter randomized, double-blind design. Central randomization process described; no major baseline imbalances reported.  Judgment: Low risk  D2: Bias due to deviations from intended interventions  Double-blind, placebo-controlled. Study drug titrated to MAP target. Conventional therapy allowed in both groups.  Judgment: Low risk  D3: Bias due to missing outcome data  Large trial (n=797). Mortality reported for all randomized patients at 28 days. Early stopping by DSMB due to harm does not introduce attrition bias.  Judgment: Low risk  D4: Bias in measurement of the outcome  28-day mortality objective.  Judgment: Low risk  D5: Bias in selection of the reported result  Primary endpoint (day-28 survival) clearly predefined. Trial stopped early for harm based on DSMB review, not selective reporting.  Judgment: Low risk  Overall: Low risk |
| Lyu 2022 | D1: Bias arising from the randomization process  Computer-generated random sequence; double-blind; allocation concealed with identical masked bags. Baseline characteristics balanced.  Judgment: Low risk  D2: Bias due to deviations from intended interventions  Double-blind. However, open-label corticosteroids allowed at clinician discretion, potentially diluting group differences. ITT analysis performed.  Judgment: Some concerns  D3: Bias due to missing outcome data  Primary analysis reported per-protocol (408/426), although ITT also presented. Exclusion from PP could introduce bias, but ITT confirmed findings.  Judgment: Some concerns  D4: Bias in measurement of the outcome  90-day mortality objective.  Judgment: Low risk  D5: Bias in selection of the reported result  Registered (NCT03872011). Primary endpoint prespecified and reported.  Judgment: Low risk  Overall: Some concerns |
| Matchett 2022 | D1: Bias arising from the randomization process  Computer-generated 1:1 sequence with sealed opaque envelopes. Block randomization used. Allocation concealment adequate.  Judgment: Low risk  D2: Bias due to deviations from intended interventions  Open-label design. Airway team not involved in subsequent ICU care. However, no blinding after randomization and possible influence on post-intubation management.  Judgment: Some concerns  D3: Bias due to missing outcome data  Data collected only after post-enrollment notification consent. Patients declining data collection excluded from analysis, introducing potential post-randomization exclusion bias.  Judgment: Some concerns  D4: Bias in measurement of the outcome  Day 7 survival objective.  Judgment: Low risk  D5: Bias in selection of the reported result  Primary endpoint prespecified and registered (NCT02643381).  Judgment: Low risk  Overall: Some concerns |
| McNamee 2021 | D1: Bias arising from the randomization process  Centralized, allocation-concealed randomization using variable block sizes, stratified by center. Baseline characteristics comparable.  Judgment: Low risk  D2: Bias due to deviations from intended interventions  Open-label pragmatic trial due to nature of ECCO₂R. Complex intervention; crossovers and co-interventions possible. ITT analysis performed.  Judgment: Some concerns  D3: Bias due to missing outcome data  405/412 (98%) completed follow-up. Minimal missing primary outcome data.  Judgment: Low risk  D4: Bias in measurement of the outcome  90-day mortality objective.  Judgment: Low risk  D5: Bias in selection of the reported result  Registered (NCT02654327). Protocol and SAP published. Early stopping for futility, but outcome reporting consistent with plan.  Judgment: Low risk  Overall: Low risk |
| Meade 2008 | D1: Bias arising from the randomization process  Central computerized telephone randomization with stratification by site and variable permuted blocks. A late programming error disrupted block balance, but sensitivity analyses confirmed preserved randomization. Baseline characteristics comparable.  Judgment: Low risk  D2: Bias due to deviations from intended interventions  Open-label ventilation strategies. Clinicians aware of allocation; co-interventions (PEEP, recruitment maneuvers, adjunct therapies) differed by design. However, primary outcome is objective and ITT analysis performed.  Judgment: Some concerns  D3: Bias due to missing outcome data  Hospital mortality available for virtually all randomized patients. No evidence of differential attrition.  Judgment: Low risk  D4: Bias in measurement of the outcome  All-cause hospital mortality objective and not susceptible to measurement bias.  Judgment: Low risk  D5: Bias in selection of the reported result  Trial registered (NCT00182195). Primary endpoint prespecified and reported accordingly.  Judgment: Low risk  Overall: Low risk |
| Mehta 2001 | D1: Bias arising from the randomization process  Computer-generated randomization at each center. Despite randomization, significant baseline imbalances (APACHE scores, liver failure, sex distribution) favored the intermittent dialysis group. Randomization process unable to explain imbalance.  Judgment: Some concerns  D2: Bias due to deviations from intended interventions  Open-label modality assignment. Crossovers allowed based on clinical criteria. Multiple supportive care elements not standardized across groups.  Judgment: Some concerns  D3: Bias due to missing outcome data  ICU mortality reported for all randomized patients in ITT analysis.  Judgment: Low risk  D4: Bias in measurement of the outcome  ICU mortality objective.  Judgment: Low risk  D5: Bias in selection of the reported result  Primary outcomes clearly defined a priori. No evidence of selective reporting.  Judgment: Low risk  Overall: Some concerns |
| Mercat 2008 | D1: Bias arising from the randomization process  Centralized interactive telephone randomization with permuted blocks stratified by center. Baseline characteristics balanced.  Judgment: Low risk  D2: Bias due to deviations from intended interventions  Open-label ventilation strategies. Co-interventions (fluid management, adjunct therapies) may have differed. ITT analysis used. Mortality objective.  Judgment: Some concerns  D3: Bias due to missing outcome data  28-day mortality available for nearly all participants; no meaningful differential loss.  Judgment: Low risk  D4: Bias in measurement of the outcome  Mortality objective and unlikely influenced by lack of blinding.  Judgment: Low risk  D5: Bias in selection of the reported result  Trial registered (NCT00188058). Primary endpoint clearly prespecified and reported.  Judgment: Low risk  Overall: Low risk |
| Meyhoff 2022 | D1: Bias arising from the randomization process  Central computerized randomization with stratification by site and cancer status; permuted blocks. Baseline characteristics balanced.  Judgment: Low risk  D2: Bias due to deviations from intended interventions  Open-label pragmatic design. Fluid administration protocol differed substantially between groups. However, ITT analysis performed and primary outcome objective.  Judgment: Some concerns  D3: Bias due to missing outcome data  Primary outcome data available for 99.4% of randomized patients. Very low and balanced loss to follow-up.  Judgment: Low risk  D4: Bias in measurement of the outcome  90-day mortality objective and not susceptible to measurement bias.  Judgment: Low risk  D5: Bias in selection of the reported result  Protocol and SAP published prior to trial completion (NCT03668236). Primary outcome reported as prespecified.  Judgment: Low risk  Overall: Low risk |
| Mohamed 2023 | D1: Bias arising from the randomization process  Randomized controlled trial; method of sequence generation described but allocation concealment not clearly detailed in available text. Small sample size and early termination may increase imbalance risk.  Judgment: Some concerns  D2: Bias due to deviations from intended interventions  Open-label design. Clinicians aware of allocation. Co-interventions not strictly standardized.  Judgment: Some concerns  D3: Bias due to missing outcome data  Small sample (n=106). No clear evidence of major missing primary outcome data, but early termination and small size increase uncertainty.  Judgment: Some concerns  D4: Bias in measurement of the outcome  In-hospital mortality objective.  Judgment: Low risk  D5: Bias in selection of the reported result  Registered (NCT03380507). Primary outcome clearly defined. Early termination for funding reasons but no evidence of selective outcome reporting.  Judgment: Low risk  Overall: Some concerns |
| Muller 2025 | D1: Bias arising from the randomization process  Centralized web-based randomization with permuted blocks of varying sizes, stratified by center, mechanical ventilation, and vasopressor dose. Allocation concealment ensured. Baseline characteristics balanced.  Judgment: Low risk  D2: Bias due to deviations from intended interventions  Open-label noninferiority design. Crossover permitted in the noninvasive group under prespecified safety criteria. However, primary analysis performed according to intention-to-treat and outcome objective.  Judgment: Some concerns  D3: Bias due to missing outcome data  28-day mortality available for virtually all randomized patients (504 vs 502 analyzed). No signal of differential attrition.  Judgment: Low risk  D4: Bias in measurement of the outcome  All-cause mortality objective and not susceptible to measurement bias.  Judgment: Low risk  D5: Bias in selection of the reported result  Registered (NCT03680963). Noninferiority margin and analysis plan prespecified. Primary outcome reported as planned.  Judgment: Low risk  Overall: Low risk |
| Myburgh 2022 | D1: Bias arising from the randomization process  Cluster, crossover randomization at ICU level using computer-generated allocation, stratified by ICU size. Randomization method appropriate.  Judgment: Low risk  D2: Bias due to deviations from intended interventions  Cluster-level intervention without individual consent; contamination minimized by ICU-wide implementation. Pragmatic design; clinicians aware of allocation. However, outcome objective.  Judgment: Some concerns  D3: Bias due to missing outcome data  All enrolled patients included in analysis; no individual loss to follow-up for mortality.  Judgment: Low risk  D4: Bias in measurement of the outcome  In-hospital mortality objective.  Judgment: Low risk  D5: Bias in selection of the reported result  Registered (NCT02389036). SAP prespecified. Primary outcome reported consistently.  Judgment: Low risk  Overall: Low risk |
| Olsen 2020 | D1: Bias arising from the randomization process  Centralized computer-generated randomization with variable block sizes, stratified by center, age, and shock. Allocation concealment ensured. Minor baseline imbalance (APACHE II slightly higher in nonsedation group).  Judgment: Low risk  D2: Bias due to deviations from intended interventions  Open-label design. Sedation strategies differed substantially; crossover not allowed. Co-interventions possible, but ITT used and mortality objective.  Judgment: Some concerns  D3: Bias due to missing outcome data  700/710 included in modified ITT; exclusions minimal and balanced. Mortality follow-up complete.  Judgment: Low risk  D4: Bias in measurement of the outcome  90-day mortality objective.  Judgment: Low risk  D5: Bias in selection of the reported result  Registered (NCT01967680). Protocol and SAP published. Primary endpoint reported as prespecified.  Judgment: Low risk  Overall: Low risk |
| Papazian 2013 | D1: Bias arising from the randomization process  Computer-generated block randomization stratified by center. Allocation concealment ensured via centralized procedure.  Judgment: Low risk  D2: Bias due to deviations from intended interventions  Double-blind, placebo-controlled design. Blinding included patients, clinicians, evaluators, and analysts.  Judgment: Low risk  D3: Bias due to missing outcome data  Trial stopped early for futility at interim analysis. No major missing outcome data reported.  Judgment: Low risk  D4: Bias in measurement of the outcome  28-day mortality objective and blinded.  Judgment: Low risk  D5: Bias in selection of the reported result  Registered (NCT01057758). Primary endpoint prespecified. Interim stopping rule predefined.  Judgment: Low risk  Overall: Low risk |
| Park 2016 | D1: Bias arising from the randomization process  Centralized computer-generated adaptive randomization. Allocation concealment described. Baseline characteristics balanced.  Judgment: Low risk  D2: Bias due to deviations from intended interventions  Open-label design. CRRT dose clearly protocolized. Co-interventions at nephrologist discretion. Mortality objective.  Judgment: Some concerns  D3: Bias due to missing outcome data  Primary outcome (28-day mortality) available for all randomized patients included in final analysis. Exclusions before analysis limited and predefined.  Judgment: Some concerns  D4: Bias in measurement of the outcome  Mortality objective.  Judgment: Low risk  D5: Bias in selection of the reported result  Registered (NCT01191905). Primary endpoint prespecified and reported.  Judgment: Low risk  Overall: Some concerns |
| Payen 2015 | D1: Bias arising from the randomization process  Centralized randomization (1:1), stratified by center with block size of 4. Allocation concealed through centralized system. Baseline characteristics comparable.  Judgment: Low risk  D2: Bias due to deviations from intended interventions  Open-label design (hemoperfusion not blindable). Standard care otherwise per Surviving Sepsis Campaign. ITT analysis performed. Mortality objective.  Judgment: Some concerns  D3: Bias due to missing outcome data  243 randomized; mortality at day 28 reported for nearly all patients. No evidence of differential loss affecting primary endpoint.  Judgment: Low risk  D4: Bias in measurement of the outcome  28-day mortality objective and not influenced by lack of blinding.  Judgment: Low risk  D5: Bias in selection of the reported result  Registered (NCT01222663). Primary endpoint prespecified and reported accordingly.  Judgment: Low risk  Overall: Low risk |
| Pettilä 2025 | D1: Bias arising from the randomization process  Computer-based randomization with stratification by site and chronic hypertension; varying block sizes. Allocation concealed.  Judgment: Low risk  D2: Bias due to deviations from intended interventions  Open-label. Complex hemodynamic strategies (TTP vs MAP-guided care) likely influenced co-interventions. Outcome includes lactate normalization and vasopressor discontinuation, both susceptible to clinician behavior.  Judgment: Some concerns  D3: Bias due to missing outcome data  194/219 analyzed for primary outcome; exclusions related to consent withdrawal. Missingness moderate but balanced between groups.  Judgment: Some concerns  D4: Bias in measurement of the outcome  Composite endpoint includes biochemical normalization and vasopressor cessation, potentially influenced by treatment strategy and clinical decision-making in an unblinded trial.  Judgment: Some concerns  D5: Bias in selection of the reported result  Protocol published; trial registered. Primary outcome clearly prespecified.  Judgment: Low risk  Overall: Some concerns |
| Rhodes 2002 | D1: Bias arising from the randomization process  Computer-generated randomization with sealed envelopes. Baseline characteristics similar.  Judgment: Low risk  D2: Bias due to deviations from intended interventions  Open-label; no management protocol mandated. Five patients randomized to PAC did not receive PAC; ITT analysis performed.  Judgment: Some concerns  D3: Bias due to missing outcome data  All randomized patients included in ITT analysis; minimal loss.  Judgment: Low risk  D4: Bias in measurement of the outcome  28-day mortality objective.  Judgment: Low risk  D5: Bias in selection of the reported result  Primary outcome clearly stated a priori; no indication of selective reporting.  Judgment: Low risk  Overall: Low risk |
| Richard 2003 | D1: Bias arising from the randomization process  Central telephone randomization using permuted blocks stratified by center. Allocation concealment appropriate.  Judgment: Low risk  D2: Bias due to deviations from intended interventions  Open-label; no standardized treatment protocol. Clinicians aware of allocation; management decisions influenced by PAC data. However, ITT used and mortality objective.  Judgment: Some concerns  D3: Bias due to missing outcome data  676 randomized; mortality follow-up complete to 28 days.  Judgment: Low risk  D4: Bias in measurement of the outcome  28-day mortality objective and unlikely influenced by lack of blinding.  Judgment: Low risk  D5: Bias in selection of the reported result  Primary endpoint clearly defined in protocol; reported as prespecified.  Judgment: Low risk  Overall: Low risk |
| Richard 2024 | D1: Bias arising from the randomization process  Randomization in permuted blocks stratified by center. Allocation concealment appropriate; analyses conducted blinded.  Judgment: Low risk  D2: Bias due to deviations from intended interventions  Open-label mechanical ventilation strategies. Sedation and spontaneous ventilation management protocolized but clinician-dependent adjustments possible. ITT analysis used.  Judgment: Some concerns  D3: Bias due to missing outcome data  700 randomized; no indication of significant loss to follow-up for day 60 mortality.  Judgment: Low risk  D4: Bias in measurement of the outcome  In-hospital mortality at day 60 objective.  Judgment: Low risk  D5: Bias in selection of the reported result  Registered (NCT01862016). Primary endpoint prespecified and reported consistently.  Judgment: Low risk  Overall: Low risk |
| Rivers 2001 | D1: Bias arising from the randomization process  Computer-generated randomization in blocks of 2–8 with sealed, opaque envelopes opened by a non-investigator. Baseline characteristics similar between groups.  Judgment: Low risk  D2: Bias due to deviations from intended interventions  Open-label during the 6-hour ED protocol; treating clinicians aware of allocation. However, ICU clinicians were blinded after admission. Primary outcome objective and ITT analysis performed.  Judgment: Some concerns  D3: Bias due to missing outcome data  In-hospital mortality available for all randomized patients. No evidence of differential loss.  Judgment: Low risk  D4: Bias in measurement of the outcome  In-hospital mortality objective.  Judgment: Low risk  D5: Bias in selection of the reported result  Primary outcome clearly prespecified; no evidence of selective reporting.  Judgment: Low risk  Overall: Low risk |
| Schefold 2014 | D1: Bias arising from the randomization process  External computer-based telephone randomization using permuted blocks of four; allocation concealment adequate. Baseline characteristics balanced.  Judgment: Low risk  D2: Bias due to deviations from intended interventions  Open-label single-center design. Switching between RRT modalities allowed for clinical reasons, introducing potential crossover effects.  Judgment: Some concerns  D3: Bias due to missing outcome data  Follow-up to 14 days after RRT reported; no major differential loss described.  Judgment: Low risk  D4: Bias in measurement of the outcome  Survival objective and not susceptible to measurement bias.  Judgment: Low risk  D5: Bias in selection of the reported result  Registered (NCT01228123). Primary endpoint prespecified and reported.  Judgment: Low risk  Overall: Some concerns |
| Schjørring 2021 | D1: Bias arising from the randomization process  Centralized concealed randomization with computer-generated sequence and stratification by site and COPD/hematologic cancer status.  Judgment: Low risk  D2: Bias due to deviations from intended interventions  Open-label due to oxygen targets. Clinicians aware of allocation; however, outcome objective and ITT analysis performed.  Judgment: Some concerns  D3: Bias due to missing outcome data  40 patients excluded post-randomization due to consent withdrawal or loss; primary analysis included 1441 and 1447 patients. Missingness low and balanced.  Judgment: Low risk  D4: Bias in measurement of the outcome  90-day mortality objective.  Judgment: Low risk  D5: Bias in selection of the reported result  Protocol and SAP published prior to trial completion; trial registered (NCT03174002).  Judgment: Low risk  Overall: Low risk |
| Semler 2018 | D1: Bias arising from the randomization process  Cluster-randomized, multiple-crossover design by ICU and month. Random allocation sequence appropriate; baseline characteristics similar.  Judgment: Low risk  D2: Bias due to deviations from intended interventions  Unblinded pragmatic design. Crossover exposure possible across calendar months; relative contraindications allowed clinician override. Potential contamination.  Judgment: Some concerns  D3: Bias due to missing outcome data  Outcome components obtained from electronic health records; minimal missing data. Mortality complete.  Judgment: Low risk  D4: Bias in measurement of the outcome  Composite includes death (objective), RRT initiation (objectively recorded), and persistent renal dysfunction (lab-based). Unblinded design may influence RRT decisions.  Judgment: Some concerns  D5: Bias in selection of the reported result  Trial registered (NCT02444988). Protocol and SAP published a priori.  Judgment: Low risk  Overall: Some concerns |
| Shapiro 2023 | D1: Bias arising from the randomization process  Central web-based randomization with stratification by site; allocation concealment adequate. Baseline characteristics balanced.  Judgment: Low risk  D2: Bias due to deviations from intended interventions  Open-label superiority trial. Protocol override permitted; clinicians aware of allocation. However, ITT analysis performed and mortality objective.  Judgment: Some concerns  D3: Bias due to missing outcome data  5 patients in restrictive group and 4 in liberal group censored due to loss to follow-up; low and balanced.  Judgment: Low risk  D4: Bias in measurement of the outcome  Death before discharge home by day 90 objective.  Judgment: Low risk  D5: Bias in selection of the reported result  Registered (NCT03434028). Protocol and SAP prespecified; outcome reported accordingly.  Judgment: Low risk  Overall: Low risk |
| Smith 2012 | D1: Bias arising from the randomization process  Central telephone/web-based randomization with computer-generated sequence and minimization by center, PaO₂/FiO₂ ratio, and age. Allocation concealment ensured; baseline characteristics comparable.  Judgment: Low risk  D2: Bias due to deviations from intended interventions  Double-blind, placebo-controlled; participants, clinicians, and investigators masked. Treatment adjustments prespecified for adverse events.  Judgment: Low risk  D3: Bias due to missing outcome data  Only one withdrawal per group; primary outcome analyzed by intention-to-treat (161 vs 163). Missing data negligible and balanced.  Judgment: Low risk  D4: Bias in measurement of the outcome  28-day mortality objective and not susceptible to assessment bias.  Judgment: Low risk  D5: Bias in selection of the reported result  Registered (ISRCTN38366450; EudraCT 2006-002647-86). Primary endpoint prespecified and reported accordingly.  Judgment: Low risk  Overall: Low risk |
| Sprung 2008 | D1: Bias arising from the randomization process  Computer-generated randomization in blocks of four, stratified by center; allocation concealed with identical numbered boxes. Baseline characteristics balanced.  Judgment: Low risk  D2: Bias due to deviations from intended interventions  Double-blind, placebo-controlled; patients, clinicians, and investigators masked. Protocolized dosing and tapering.  Judgment: Low risk  D3: Bias due to missing outcome data  499 randomized; mortality reported for all randomized patients; ITT analysis conducted.  Judgment: Low risk  D4: Bias in measurement of the outcome  28-day mortality objective.  Judgment: Low risk  D5: Bias in selection of the reported result  Trial registered (NCT00147004). Primary endpoint clearly prespecified (28-day mortality among non-responders).  Judgment: Low risk  Overall: Low risk |
| Stephens 2008 | D1: Bias arising from the randomization process  Computer-generated block randomization held by pharmacy; allocation concealment ensured with identical syringes. Baseline severity similar.  Judgment: Low risk  D2: Bias due to deviations from intended interventions  Double-blind; ICU staff unaware of allocation. Septic shock management protocol standardized across groups.  Judgment: Low risk  D3: Bias due to missing outcome data  166 randomized; primary ITT analysis included all patients with available data. No major attrition affecting mortality endpoint.  Judgment: Low risk  D4: Bias in measurement of the outcome  Hospital mortality objective.  Judgment: Low risk  D5: Bias in selection of the reported result  Primary endpoint prespecified; no evidence of selective reporting.  Judgment: Low risk  Overall: Low risk |
| Taccone 2009 | D1: Bias arising from the randomization process  Centralized telephone randomization with computer-generated permuted blocks, stratified by severity and center. Allocation concealment appropriate.  Judgment: Low risk  D2: Bias due to deviations from intended interventions  Unblinded design; clinicians aware of prone vs supine assignment. However, ventilation protocol standardized and mortality objective. ITT analysis performed.  Judgment: Some concerns  D3: Bias due to missing outcome data  342 randomized; primary endpoint reported for all. No significant differential loss.  Judgment: Low risk  D4: Bias in measurement of the outcome  28-day mortality objective and unlikely influenced by lack of blinding.  Judgment: Low risk  D5: Bias in selection of the reported result  Registered (NCT00159939). Primary endpoint prespecified and reported as planned.  Judgment: Low risk  Overall: Low risk |
| Tongyoo 2016 | D1: Bias arising from the randomization process  Computer-generated randomization table; allocation prepared by independent research nurse; double-blind design. Baseline characteristics balanced.  Judgment: Low risk  D2: Bias due to deviations from intended interventions  Double-blind, placebo-controlled; management per Surviving Sepsis and ARDS guidelines. Minimal risk of performance bias.  Judgment: Low risk  D3: Bias due to missing outcome data  197 randomized; ITT analysis performed; minimal exclusions after randomization; flow diagram shows balanced attrition.  Judgment: Low risk  D4: Bias in measurement of the outcome  28-day mortality objective.  Judgment: Low risk  D5: Bias in selection of the reported result  Registered (NCT01284452). Primary endpoint prespecified and reported accordingly.  Judgment: Low risk  Overall: Low risk |
| Van den Berghe 2001 | D1: Bias arising from the randomization process  Randomization by sealed envelopes with permuted blocks of 10, stratified by type of critical illness. Baseline characteristics well balanced. However, envelope-based allocation carries potential risk if not fully safeguarded.  Judgment: Some concerns  D2: Bias due to deviations from intended interventions  Open-label glycemic management; clinicians aware of group assignment. Glucose management protocolized, but co-interventions could differ. Primary outcome objective; ITT analysis performed.  Judgment: Some concerns  D3: Bias due to missing outcome data  Mortality during ICU stay reported for all randomized patients; no meaningful missing data for primary endpoint.  Judgment: Low risk  D4: Bias in measurement of the outcome  ICU mortality objective and not susceptible to measurement bias.  Judgment: Low risk  D5: Bias in selection of the reported result  Primary endpoint prespecified; outcomes reported consistently with protocol.  Judgment: Low risk  Overall: Some concerns |
| van der Wal 2023 | D1: Bias arising from the randomization process  Secure web-based randomization with variable block sizes, stratified by site. Allocation concealment appropriate. Baseline characteristics balanced.  Judgment: Low risk  D2: Bias due to deviations from intended interventions  Open-label oxygenation targets; clinicians not blinded. Oxygen titration directly influenced by allocation. However, mortality objective and ITT analysis used.  Judgment: Some concerns  D3: Bias due to missing outcome data  28-day mortality available for all randomized patients included in analysis; no important differential loss.  Judgment: Low risk  D4: Bias in measurement of the outcome  28-day mortality objective.  Judgment: Low risk  D5: Bias in selection of the reported result  Trial prospectively registered (NTR7376). Primary endpoint clearly prespecified and reported.  Judgment: Low risk  Overall: Low risk |
| Wacker 2022 | D1: Bias arising from the randomization process  Site-stratified randomization; double-blind design; allocation concealment described. Baseline characteristics balanced.  Judgment: Low risk  D2: Bias due to deviations from intended interventions  Double-blind, placebo-controlled. Minimal deviation risk. ITT analysis performed.  Judgment: Low risk  D3: Bias due to missing outcome data  124 patients analyzed; minimal withdrawal after randomization; outcome data complete for primary endpoint.  Judgment: Low risk  D4: Bias in measurement of the outcome  28-day mortality objective and blinded.  Judgment: Low risk  D5: Bias in selection of the reported result  Registered (NCT03338569). Primary outcome prespecified and reported accordingly.  Judgment: Low risk  Overall: Low risk |
| Warren 2001 | D1: Bias arising from the randomization process  Central randomization with block size of 4; allocation through independent randomization center. Baseline comparability reported.  Judgment: Low risk  D2: Bias due to deviations from intended interventions  Double-blind, placebo-controlled. However, concomitant heparin use allowed and interacted with treatment effect, introducing potential performance heterogeneity.  Judgment: Some concerns  D3: Bias due to missing outcome data  Primary efficacy population included randomized patients who received study drug and had known survival at 28 days; minimal missing data.  Judgment: Low risk  D4: Bias in measurement of the outcome  28-day mortality objective and blinded.  Judgment: Low risk  D5: Bias in selection of the reported result  Protocol prespecified primary endpoint and stratified analysis; reporting consistent.  Judgment: Low risk  Overall: Low risk |
| Wheeler 2006 | D1: Bias arising from the randomization process  Centralized randomization with permuted blocks and stratification by site and fluid strategy. Allocation concealment appropriate. Baseline characteristics similar.  Judgment: Low risk  D2: Bias due to deviations from intended interventions  Open-label catheter assignment. Management strictly protocolized. Very low crossover rate (~1%). Primary outcome objective.  Judgment: Some concerns  D3: Bias due to missing outcome data  Follow-up for primary endpoint complete; assumption of survival at 60 days for those discharged home prespecified. Missing data minimal.  Judgment: Low risk  D4: Bias in measurement of the outcome  Mortality objective and not influenced by lack of blinding.  Judgment: Low risk  D5: Bias in selection of the reported result  ClinicalTrials registration (NCT00281268); primary outcome prespecified and reported accordingly.  Judgment: Low risk  Overall: Low risk |
| Wolfrum 2022 | D1: Bias arising from the randomization process  Randomization performed centrally via hotline using permuted blocks of 10, stratified by center. Block size concealed. Baseline characteristics comparable.  Judgment: Low risk  D2: Bias due to deviations from intended interventions  Open-label temperature management. Treating clinicians aware of allocation; however, outcome assessors and analysts were blinded. Primary outcome objective (mortality). ITT analysis performed.  Judgment: Some concerns  D3: Bias due to missing outcome data  Mortality at day 180 reported for nearly all randomized patients (87/120 vs 84/118 analyzed). No signal of differential attrition.  Judgment: Low risk  D4: Bias in measurement of the outcome  All-cause mortality objective and assessed blinded to allocation.  Judgment: Low risk  D5: Bias in selection of the reported result  Registered (NCT00457431). Primary endpoint prespecified. Trial stopped early for futility but according to monitoring plan.  Judgment: Low risk  Overall: Low risk |
| Young 2013 | D1: Bias arising from the randomization process  Central automated telephone randomization using minimization algorithm with probabilistic allocation (80%) balancing key covariates. Appropriate allocation concealment.  Judgment: Low risk  D2: Bias due to deviations from intended interventions  Open-label. Significant non-adherence: 91.9% received early tracheostomy vs 44.9% in late group. However, ITT analysis performed and mortality objective.  Judgment: Some concerns  D3: Bias due to missing outcome data  Primary mortality outcome complete for all randomized patients. Vital status obtained via national registry.  Judgment: Low risk  D4: Bias in measurement of the outcome  30-day mortality objective.  Judgment: Low risk  D5: Bias in selection of the reported result  Registered (ISRCTN28588190). Primary endpoint prespecified and reported accordingly.  Judgment: Low risk  Overall: Low risk |
| Young 2020 | D1: Bias arising from the randomization process  Cluster crossover randomization stratified by region and time period. Appropriate computer-generated allocation.  Judgment: Low risk  D2: Bias due to deviations from intended interventions  Open-label cluster design with substantial crossover (≈20% in H2RB group received PPIs). Treatment adherence incomplete and patient-level exposure not fully captured.  Judgment: Some concerns  D3: Bias due to missing outcome data  26 771/26 828 (99.2%) included in mortality analysis. Minimal and balanced missing data.  Judgment: Low risk  D4: Bias in measurement of the outcome  In-hospital mortality objective and registry-based.  Judgment: Low risk  D5: Bias in selection of the reported result  Primary outcome changed during trial from composite to mortality (March 2017). Change documented and justified, but occurred after trial initiation.  Judgment: Some concerns  Overall: Some concerns |
| Zampieri 2021 | D1: Bias arising from the randomization process  Central web-based randomization using permuted blocks stratified by center and factorial allocation. Allocation concealment appropriate.  Judgment: Low risk  D2: Bias due to deviations from intended interventions  Unblinded. Clinicians aware of infusion rate; faster rates allowed at physician discretion in some cases. However, ITT analysis performed and mortality objective.  Judgment: Some concerns  D3: Bias due to missing outcome data  10 520/11 052 (95.2%) analyzed; exclusions limited to duplicates and consent withdrawals. Mortality follow-up complete.  Judgment: Low risk  D4: Bias in measurement of the outcome  90-day mortality objective.  Judgment: Low risk  D5: Bias in selection of the reported result  Registered (NCT02875873). Primary endpoint prespecified and reported as planned.  Judgment: Low risk  Overall: Low risk |
| Zarbock 2016 | D1: Bias arising from the randomization process  Randomized single-center trial; allocation method described but limited detail on concealment beyond central registration. Baseline comparable.  Judgment: Some concerns  D2: Bias due to deviations from intended interventions  Unblinded timing strategy. All early patients received RRT; 90.8% in delayed group received RRT. Possible performance bias. ITT analysis performed.  Judgment: Some concerns  D3: Bias due to missing outcome data  Complete 90-day follow-up for all patients.  Judgment: Low risk  D4: Bias in measurement of the outcome  90-day mortality objective.  Judgment: Low risk  D5: Bias in selection of the reported result  Registered (DRKS00004367). Primary endpoint prespecified.  Judgment: Low risk  Overall: Some concerns |
| Zarbock 2020 | D1: Bias arising from the randomization process  Centralized randomization across 26 centers; allocation concealment adequate. Baseline characteristics balanced.  Judgment: Low risk  D2: Bias due to deviations from intended interventions  Open-label anticoagulation strategy. Protocolized management but clinicians aware of assignment. Coprimary endpoint includes mortality (objective). ITT used.  Judgment: Some concerns  D3: Bias due to missing outcome data  596/638 completed trial; minimal missing for mortality endpoint.  Judgment: Low risk  D4: Bias in measurement of the outcome  90-day mortality objective.  Judgment: Low risk  D5: Bias in selection of the reported result  Registered (NCT02669589). Coprimary endpoints prespecified. Trial stopped early, potentially underpowered for mortality.  Judgment: Some concerns  Overall: Some concerns |

# **Supplementary Figure 1 – Risk of Bias**


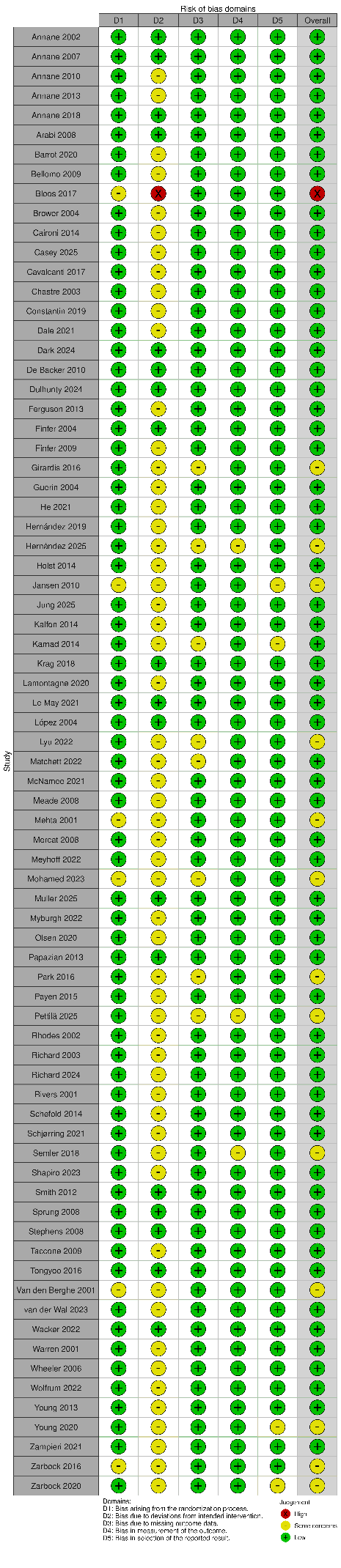


# **Supplementary Table 1 – Domain comparison 2000-2019 vs 2020-2025**

| Domain | 2000-2019 | 2020-2025 |
| --- | --- | --- |
| Hemodynamic & Resuscitation | 15 (25.4%) | 6 (22.2%) |
| Mechanical Ventilation & Oxygenation | 16 (27.1%) | 4 (14.8%) |
| Pharmacologic & Immunomodulatory Therapies | 14 (23.7%) | 7 (25.9%) |
| Renal & Metabolic Support | 9 (15.3%) | 3 (11.1%) |
| General ICU Management & Miscellaneous | 5 (8.5%) | 7 (25.9%) |

# **Table 1 – Complete Version**

| **Author** | **Year** | **Nº of patients** | **Female,**  **n (%)** | **Age, years (mean)** | **ICU population** | **Type of intervention** | **Intervention impact on mortality** | **Inclusion of oncologic patients I vs C, n (%)** |
| --- | --- | --- | --- | --- | --- | --- | --- | --- |
| Annane | 2002 | 299 | 99 (33.1) | 61 | Septic shock | Pharmacologic & Immunomodulatory Therapies | Decreased | Cancer  23 (15) vs 18 (12) |
| Annane | 2007 | 330 | 128 (28) | 63 | Septic shock | Hemodynamic & Resuscitation | Neutral | Excluded |
| Annane | 2010 | 509 | 179 (35.2) | 64 | Septic shock | Pharmacologic & Immunomodulatory Therapies | Neutral | Not reported |
| Annane | 2013 | 2,857 | 1,075 (37.6) | 62.8 | Hypovolemic shock | Hemodynamic & Resuscitation | Neutral | Not reported |
| Annane | 2018 | 1,241 | 414 (33.4) | 66 | Septic shock | Pharmacologic & Immunomodulatory Therapies | Decreased | Not reported |
| Arabi | 2008 | 523 | 132 (25.2) | 52.4 | Medical and surgical | Renal & Metabolic Support | Neutral | Not reported |
| Barrot | 2020 | 201 | 72 (35.8) | 63.3 | ARDS | Mechanical Ventilation & Oxygenation | Neutral | Cancer  24 (24.2) vs 20 (19.6) |
| Bellomo | 2009 | 1,465 | 519 (35.4) | 64.5 | AKI | Renal & Metabolic Support | Neutral | Not reported |
| Bloss | 2017 | 4,183 | 1,572 (37.6) | 70 | Septic shock | General ICU Management & Miscellaneous | Neutral | Not reported |
| Brower | 2004 | 549 | 247 (45) | 51.1 | ARDS | Mechanical Ventilation & Oxygenation | Neutral | Not reported |
| Busund | 2002 | 106 | 46 (43.4) | 44.4 | Septic shock | Pharmacologic & Immunomodulatory Therapies | Decreased | Excluded |
| Caironi | 2014 | 1,819 | 717 (39.6) | 68.2 | Septic shock | Hemodynamic & Resuscitation | Neutral | Not reported |
| Casey | 2025 | 2,365 | 990 (41.8) | 60 | Mixed population | Pharmacologic & Immunomodulatory Therapies | Neutral | Cancer  227 (19.3) vs 215 (18.1) |
| Cavalcanti | 2017 | 1,010 | 379 (37.5) | 50.9 | ARDS | Mechanical Ventilation & Oxygenation | Increased | Not reported |
| Chastre | 2003 | 401 | 112 (27.9) | 60.5 | VAP | Pharmacologic & Immunomodulatory Therapies | Neutral | Excluded |
| Constantin | 2019 | 400 | 124 (31) | 62 | ARDS | Mechanical Ventilation & Oxygenation | Neutral | Haematological cancer  10 (5) vs 6 (3) Solid cancer  4 (2) vs 1 (1) |
| Dale | 2021 | 3,260 | 1,248 (38.3) | 59.8 | General ICU | General ICU Management & Miscellaneous | Neutral | Not reported |
| Dark | 2025 | 2,748 | 1,091 (39.7) | 60.2 | Sepsis | Pharmacologic & Immunomodulatory Therapies | Neutral | Not reported |
| De Backer | 2010 | 1,679 | 723 (43.1) | 66.3 | Shock | Hemodynamic & Resuscitation | Neutral | Not reported |
| Dulhunty | 2024 | 7,031 | 2,423 (34.4) | 59.5 | Sepsis | Pharmacologic & Immunomodulatory Therapies | Neutral | Not reported |
| Ferguson | 2013 | 548 | 228 (41.6) | 54.5 | ARDS | Mechanical Ventilation & Oxygenation | Increased | Not reported |
| Finfer | 2004 | 6,997 | 2,800 (40) | 58.5 | Mixed population | Hemodynamic & Resuscitation | Neutral | Not reported |
| Finfer | 2009 | 6,030 | 2,207 (36.6) | 60.2 | Mixed population | Renal & Metabolic Support | Increased | Not reported |
| Girardis | 2016 | 434 | 188 (43.3) | 63.5 | Mixed population | Mechanical Ventilation & Oxygenation | Decreased | Cancer 72 (33.3) vs 70 (31.1) |
| Guerin | 2004 | 791 | 198 (25) | 62.2 | ARF | Mechanical Ventilation & Oxygenation | Neutral | Included, but data unavaliable |
| Guerin | 2013 | 466 | 148 (31.8) | 59 | ARDS | Mechanical Ventilation & Oxygenation | Decreased | Cancer 30 (13.1) vs 24 (10.1) |
| He | 2021 | 117 | 40 (34.18) | 60.3 | ARDS | Mechanical Ventilation & Oxygenation | Neutral | Not reported |
| Hernandez | 2019 | 424 | 226 (53.3) | 63 | Septic shock | Hemodynamic & Resuscitation | Neutral | Not reported |
| Hernandez | 2025 | 1,467 | 636 (43.3) | 66 | Septic shock | Hemodynamic & Resuscitation | Neutral | Nonhematological cancer 46 (6.6) vs 30 (4.1)  Hematological cancer  12 (1.7) vs 21 (2.9) |
| Holst | 2014 | 998 | 467 (46.8) | 67 | Septic shock | Hemodynamic & Resuscitation | Neutral | Not reported |
| Jansen | 2010 | 348 | 127 (36.5) | 62 | Mixed population | Hemodynamic & Resuscitation | Decreased | Not reported |
| Jung | 2025 | 627 | 248 (39.5) | 67 | AKI + Severe metabolic acidemia | Renal & Metabolic Support | Neutral | Not reported |
| Kalfon | 2014 | 2,648 | 942 (35.6) | 61.5 | Mixed population | Renal & Metabolic Support | Neutral | Hematologic or solid tumor  9 (0.7) vs 7 (0.5) |
| Karnad | 2014 | 114 | 26 (22.8) | 37.1 | Sepsis | Pharmacologic & Immunomodulatory Therapies | Decreased | Excluded |
| Krag | 2018 | 3,298 | 1,185 (36) | 67 | Mixed population | Pharmacologic & Immunomodulatory Therapies | Neutral | Included metastatic cancer/hematologic cancer: 56(3%)/64(4%) x 55 (3%)/55(3%) |
| Lamontagne | 2020 | 2,455 | 1,067 (43.5) | 75.3 | Vasodilatory shock | Hemodynamic & Resuscitation | Neutral | Not reported |
| Le May | 2021 | 367 | 69 (18.8) | 61.3 | Comatose OHCA | General ICU Management & Miscellaneous | Neutral | Not reported |
| Lopez | 2004 | 797 | 305 (38.3) | 64 | Septic shock | Pharmacologic & Immunomodulatory Therapies | Increased | Cancer 38 (9) x 39 (11) |
| Lyu | 2022 | 426 | 141 (33.1) | 69.5 | Septic shock | Pharmacologic & Immunomodulatory Therapies | Neutral | Malignancy  32 (15) vs 41 (19.2) |
| Matchett | 2022 | 791 | 303 (38.3) | 55.6 | Mixed population | General ICU Management & Miscellaneous | Neutral | Not reported |
| McNamee | 2021 | 412 | 143 (34.7) | 60.3 | ARDS | Mechanical Ventilation & Oxygenation | Neutral | Not reported |
| Meade | 2008 | 983 | 394 (40.1) | 55.7 | ARDS | Mechanical Ventilation & Oxygenation | Neutral | Not reported |
| Mehta | 2001 | 166 | 40 (24.1) | 55.4 | ARF | Renal & Metabolic Support | Neutral | Not reported |
| Mercat | 2008 | 767 | 251 (32.7) | 59.9 | ARDS | Mechanical Ventilation & Oxygenation | Neutral | Not reported |
| Meyhoff | 2022 | 1,531 | 627 (40.9) | 70.5 | Septic shock | Hemodynamic & Resuscitation | Neutral | Hematologic or metastatic cancer 128 (17.0) vs 140 (18.0) |
| Mohamed | 2023 | 106 | 31 (29.2) | 49.2 | Septic shock | Pharmacologic & Immunomodulatory Therapies | Neutral | Not reported |
| Muller | 2025 | 1,006 | 324 (32.2) | 66 | Shock | General ICU Management & Miscellaneous | Neutral | Active solid-organ cancer  45 (8.9) vs 47 (9.4)  Active hematologic cancer  29 (5.8) vs 47 (9.4) |
| Myburgh | 2022 | 5,982 | 2,202 (36.8) | 58.3 | Mechanical ventilation | General ICU Management & Miscellaneous | Neutral | Not reported |
| Olsen | 2020 | 700 | 273 (39) | 71 | Mechanical ventilation | General ICU Management & Miscellaneous | Neutral | Not reported |
| Papazian | 2013 | 284 | 71 (25) | 59 | VAP | Pharmacologic & Immunomodulatory Therapies | Neutral | Not reported |
| Park | 2016 | 212 | 74 (34.9) | 62.1 | Sepsis + AKI | Renal & Metabolic Support | Neutral | Excluded terminal cancer only; other cancer patients not reported |
| Payen | 2015 | 232 | 98 (42.2) | 71.7 | Septic shock | General ICU Management & Miscellaneous | Neutral | Cancer or hematological malignany  35 (30) vs 33 (29) |
| Petilla | 2025 | 194 | 113 (58.2) | 62 | Septic shock | Hemodynamic & Resuscitation | Neutral | Hematological malignancy  3 (3) vs 2 (2)  Metastatic cancer  6 (6) vs 7 (7) |
| Rhodes | 2002 | 201 | NA | 65.6 | Mixed population | Hemodynamic & Resuscitation | Neutral | Not reported |
| Richard | 2003 | 676 | 224 (33.1) | 62.7 | ARDS | Hemodynamic & Resuscitation | Neutral | Not reported |
| Richard | 2024 | 699 | 218 (31.2) | 62 | ARDS | Mechanical Ventilation & Oxygenation | Neutral | Not reported |
| Rivers | 2001 | 263 | 130 (49.4) | 65.7 | Septic shock | Hemodynamic & Resuscitation | Decreased | Excluded |
| Schefold | 2014 | 250 | 94 (37.6) | 61.55 | AKI | Renal & Metabolic Support | Neutral | Solid/hematologic  17/22 (14/18) vs 30/23 (23.4/17.9) |
| Schjørring | 2021 | 2,910 | 1,039 (35.7) | 70 | ARF | Mechanical Ventilation & Oxygenation | Neutral | Metastatic/hematologic  65/82 (4.5/5.6) vs 61/86 (4.2/5.9) |
| Semler | 2018 | 15,802 | 6,705 (42.4) | 58 | Mixed population | Hemodynamic & Resuscitation | Decreased | Not reported |
| Shapiro | 2023 | 1,563 | 737 (47.2) | 59.5 | Sepsis | Hemodynamic & Resuscitation | Neutral | Not reported |
| Smith | 2012 | 326 | 114 (35) | 55 | ARDS | General ICU Management & Miscellaneous | Neutral | Not reported |
| Sprung | 2008 | 499 | 167 (33.5) | 63 | Septic shock | Pharmacologic & Immunomodulatory Therapies | Neutral | Cancer  47 (19) vs 37 (15) |
| Stephens | 2008 | 164 | 75 (45.7) | 49.9 | Septic shock | Pharmacologic & Immunomodulatory Therapies | Neutral | Excluded |
| Taccone | 2009 | 342 | 98 (28.7) | 60 | ARDS | Mechanical Ventilation & Oxygenation | Neutral | Not reported |
| Tongyoo | 2016 | 197 | 96 (48.7) | 64.4 | Sepsis + ARDS | Pharmacologic & Immunomodulatory Therapies | Neutral | Cancer and/or  immunosuppression  18 (18.4) vs 26 (26.3) |
| Van den Berghe | 2001 | 1,548 | 447 (28.9) | 62.8 | Mixed population | Renal & Metabolic Support | Decreased | Cancer  122 (16) vs 119 (15) |
| Van der Wal | 2023 | 664 | 229 (34.5) | 67 | Mechanical ventilation | Mechanical Ventilation & Oxygenation | Neutral | Metastatic/hematologic  8/14 (2.4/4.2) vs 5/19 (1.5/5.8) |
| Wacker | 2022 | 124 | 61 (49.2) | 70.9 | Septic shock | Pharmacologic & Immunomodulatory Therapies | Neutral | Not reported |
| Warren | 2001 | 2,314 | 891 (38.5) | 57.5 | Sepsis | Pharmacologic & Immunomodulatory Therapies | Neutral | Excluded |
| Wheeler | 2006 | 1,000 | 467 (46.7) | 49.7 | ARDS | Hemodynamic & Resuscitation | Neutral | Solid tumor/hematologic  7/21 (1/4) vs 8/14 (2/3) |
| Wolfrum | 2022 | 238 | 86 (36.1) | 72.6 | IHCA | General ICU Management & Miscellaneous | Neutral | Not reported |
| Young | 2013 | 899 | 372 (41.4) | 63.9 | Mechanical ventilation | General ICU Management & Miscellaneous | Neutral | Not reported |
| Young | 2020 | 26,828 | 9,691 (36.1) | 58.4 | Mechanical ventilation | Pharmacologic & Immunomodulatory Therapies | Neutral | Metastatic cancer  367(2.7) vs 340 (2.5) |
| Zampieri | 2021 | 10,520 | 4,655 (44.2) | 61.1 | Mixed population | Hemodynamic & Resuscitation | Neutral | Not reported |
| Zarbock | 2016 | 231 | 85 (36.8) | 67 | AKI | Renal & Metabolic Support | Decreased | Excluded |
| Zarbock | 2020 | 596 | 183 (30.7) | 67.5 | AKI | Renal & Metabolic Support | Neutral | Not reported |

# **PRISMA CHECKLIST**

| **Topic** | **No.** | **Item** | **Location where item is reported** |
| --- | --- | --- | --- |
| **TITLE** |  |  |  |
| **Title** | 1 | Identify the report as a systematic review. | Pg. 1; MS |
| **ABSTRACT** |  |  |  |
| **Abstract** | 2 | See the PRISMA 2020 for Abstracts checklist | Pg. 2; MS |
| **INTRODUCTION** |  |  |  |
| **Rationale** | 3 | Describe the rationale for the review in the context of existing knowledge. | Pg. 3; MS |
| **Objectives** | 4 | Provide an explicit statement of the objective(s) or question(s) the review addresses. | Pg. 3; MS |
| **METHODS** |  |  |  |
| **Eligibility criteria** | 5 | Specify the inclusion and exclusion criteria for the review and how studies were grouped for the syntheses. | Pg. 4; MS |
| **Information sources** | 6 | Specify all databases, registers, websites, organizations, reference lists and other sources searched or consulted to identify studies. Specify the date when each source was last searched or consulted. | Pg. 4; MS |
| **Search strategy** | 7 | Present the full search strategies for all databases, registers, and websites, including any filters and limits used. | Pg. 4; Supp |
| **Selection process** | 8 | Specify the methods used to decide whether a study met the inclusion criteria of the review, including how many reviewers screened each record and each report retrieved, whether they worked independently, and if applicable, details of automation tools used in the process. | Pg. 4; MS |
| **Data collection process** | 9 | Specify the methods used to collect data from reports, including how many reviewers collected data from each report, whether they worked independently, any processes for obtaining or confirming data from study investigators, and if applicable, details of automation tools used in the process. | Pg. 4; MS |
| **Data items** | 10a | List and define all outcomes for which data were sought. Specify whether all results that were compatible with each outcome domain in each study were sought (e.g., for all measures, time points, analyses), and if not, the methods used to decide which results to collect. | Pg. 4; MS |
|  | 10b | List and define all other variables for which data were sought (e.g., participant and intervention characteristics, funding sources). Describe any assumptions made about any missing or unclear information. | NA |
| **Study risk of bias assessment** | 11 | Specify the methods used to assess risk of bias in the included studies, including details of the tool(s) used, how many reviewers assessed each study and whether they worked independently, and if applicable, details of automation tools used in the process. | NA |
| **Effect measures** | 12 | Specify for each outcome the effect measure(s) (e.g., risk ratio, mean difference) used in the synthesis or presentation of results. | NA |
| **Synthesis methods** | 13a | Describe the processes used to decide which studies were eligible for each synthesis (e.g., tabulating the study intervention characteristics and comparing against the planned groups for each synthesis (item 5)). | Table 1 |
|  | 13b | Describe any methods required to prepare the data for presentation or synthesis, such as handling of missing summary statistics, or data conversions. | NA. |
|  | 13c | Describe any methods used to tabulate or visually display results of individual studies and syntheses. | NA |
|  | 13d | Describe any methods used to synthesize results and provide a rationale for the choice(s). If meta-analysis was performed, describe the model(s), method(s) to identify the presence and extent of statistical heterogeneity, and software package(s) used. | NA |
|  | 13e | Describe any methods used to explore possible causes of heterogeneity among study results (e.g., subgroup analysis, meta-regression). | NA |
|  | 13f | Describe any sensitivity analyses conducted to assess robustness of the synthesized results. | NA |
| **Reporting bias assessment** | 14 | Describe any methods used to assess risk of bias due to missing results in a synthesis (arising from reporting biases). | NA |
| **Certainty assessment** | 15 | Describe any methods used to assess certainty (or confidence) in the body of evidence for an outcome. | NA |
| **RESULTS** |  |  |  |
| **Study selection** | 16a | Describe the results of the search and selection process, from the number of records identified in the search to the number of studies included in the review, ideally using a flow diagram. | Figure 1 |
|  | 16b | Cite studies that might appear to meet the inclusion criteria, but which were excluded, and explain why they were excluded. | NA |
| **Study characteristics** | 17 | Cite each included study and present its characteristics. | Table 1 |
| **Risk of bias in studies** | 18 | Present assessments of risk of bias for each included study. | NA |
| **Results of individual studies** | 19 | For all outcomes, present, for each study: (a) summary statistics for each group (where appropriate) and (b) an effect estimate and its precision (e.g., confidence/credible interval), ideally using structured tables or plots. | NA |
| **Results of syntheses** | 20a | For each synthesis, briefly summarize the characteristics and risk of bias among contributing studies. | NA |
|  | 20b | Present results of all statistical syntheses conducted. If meta-analysis was done, present for each the summary estimate and its precision (e.g., confidence/credible interval) and measures of statistical heterogeneity. If comparing groups, describe the direction of the effect. | NA |
|  | 20c | Present results of all investigations of possible causes of heterogeneity among study results. | NA |
|  | 20d | Present results of all sensitivity analyses conducted to assess the robustness of the synthesized results. | NA |
| **Reporting biases** | 21 | Present assessments of risk of bias due to missing results (arising from reporting biases) for each synthesis assessed. | NA |
| **Certainty of evidence** | 22 | Present assessments of certainty (or confidence) in the body of evidence for each outcome assessed. | NA |
| **DISCUSSION** |  |  |  |
| **Discussion** | 23a | Provide a general interpretation of the results in the context of other evidence. | Pg. 6-9; MS |
|  | 23b | Discuss any limitations of the evidence included in the review. | Pg. 6-9; MS |
|  | 23c | Discuss any limitations of the review processes used. | Pg. 6-9; MS |
|  | 23d | Discuss implications of the results for practice, policy, and future research. | Pg. 9; MS |
| **OTHER INFORMATION** |  |  |  |
| **Registration and protocol** | 24a | Provide registration information for the review, including register name and registration number, or state that the review was not registered. | [CRD420251129171](https://www.crd.york.ac.uk/PROSPERO/view/CRD420251129171) |
|  | 24b | Indicate where the review protocol can be accessed, or state that a protocol was not prepared. | <https://www.crd.york.ac.uk/PROSPERO/view/CRD420251129171>. |
|  | 24c | Describe and explain any amendments to information provided at registration or in the protocol. | NA |
| **Support** | 25 | Describe sources of financial or non-financial support for the review, and the role of the funders or sponsors in the review. | None |
| **Competing interests** | 26 | Declare any competing interests of review authors. | None |
| **Availability of data, code and other materials** | 27 | Report which of the following are publicly available and where they can be found template data collection forms; data extracted from included studies; data used for all analyses; analytic code; any other materials used in the review. | NA |
